# CDK7 inhibitor THZ1 enhances antiPD-1 therapy efficacy via the p38α/MYC/PD-L1 signaling in non-small cell lung cancer

**DOI:** 10.1186/s13045-020-00926-x

**Published:** 2020-07-20

**Authors:** Jian Wang, Ruiguang Zhang, Zhenyu Lin, Sheng Zhang, Yaobing Chen, Jing Tang, Jiaxin Hong, Xiaoshu Zhou, Yan Zong, Yingzhuo Xu, Rui Meng, Shuangbing Xu, Li Liu, Tao Zhang, Kunyu Yang, Xiaorong Dong, Gang Wu

**Affiliations:** 1 Cancer Center, Union Hospital, Tongji Medical College, Huazhong University of Science and Technology, Wuhan, 430022 China; 2 Institute of Pathology, Tongji Hospital, Tongji Medical College, Huazhong University of Science and Technology, Wuhan, 430030 China

**Keywords:** CDK7, p38α, MYC, PD-L1, Non-small cell lung cancer

## Abstract

**Background:**

The cyclin-dependent kinase 7 (CDK7) subunit of TFIIH regulates RNA polymerase-II-based transcription and promotes tumor progression. However, the mechanisms involved in CDK7-mediated immune evasion are unclear in non-small cell lung cancer (NSCLC).

**Methods:**

RNA silencing and pharmacologic inhibitors were used to evaluate the functions of CDK7/p38α/MYC/PD-L1 axis in cancer cell proliferation and antiPD-1 therapy resistance. Flow cytometry was performed to detect the status of the immune microenvironment after CDK7 inhibition and antiPD-1 therapy in vivo. CD8 depletion antibodies were used to assess the role of CD8^+^ T cells in combined CDK7 and PD-1 blockade. The associations among CDK7, p38α, MYC, PD-L1, infiltrating T cells, and survival outcomes were validated in two tissue microarrays and public transcriptomic data of NSCLC.

**Results:**

High CDK7 mRNA and protein levels were identified to be associated with poor prognosis in NSCLC. CDK7 silencing and CDK7 inhibitor THZ1 elicited apoptosis and suppressed tumor growth. Moreover, CDK7 ablation specifically suppressed p38α/MYC-associated genes, and THZ1 inhibited MYC transcriptional activity through downregulating p38α. CDK7 inhibition sensitized NSCLC to p38α inhibitor. Further, THZ1 suppressed PD-L1 expression by inhibiting MYC activity. THZ1 boosted antitumor immunity by recruiting infiltrating CD8^+^ T cells and synergized with antiPD-1 therapy. The CDK7/MYC/PD-L1 signature and infiltrating T cell status collectively stratified NSCLC patients into different risk groups.

**Conclusion:**

These data suggest that the combined CDK7 inhibitor THZ1 and antiPD-1 therapy can be an effective treatment in NSCLC.

## Introduction

Lung cancer remains the most commonly diagnosed cancer and the leading cause of cancer-related death globally [1]. Non-small cell lung cancer (NSCLC), which shows histological, genetic, and molecular heterogeneity [2], is the major form of primary lung cancer [3]. Most patients with NSCLC present with metastatic disease at diagnosis and exhibit poor prognosis and high relapse after treatment. Recently, immune checkpoint blockade therapies (ICBs) targeting PD-1/PD-L1 axis have exhibited significant clinical benefits. However, the relatively low response rate and antiPD-1 therapy resistance [4–6] highlight the need to understand the regulation of PD-L1.

The general transcription factor TFIIH is recruited to start the transition from transcription initiation to early elongation, which is a key step for transcription by RNA polymerase II (Pol II) [7–9]. TFIIH, which plays a fundamental role in transcription and DNA repair [10], is a 10-subunit complex. Seven subunits of TFIIH (p34, p44, p52, p62, XPD, XPB, and TTDA) form the core complex, and the left three form the cyclin-activating kinase complex (CAK) which consists of MAT1 and cyclin H and cyclin-dependent kinase 7 (CDK7) [8, 11]. Targeting TFIIH subunits, such as CDK7, has been a promising strategy in cancer treatment. CDK7 phosphorylates the C-terminal domain (CTD) of the pol II subunit RPB1 at serine 2, 5, and 7, which is dispensable for productive transcription elongation [12–14]. More recently, THZ1, a selective CDK7 covalent inhibitor, has been shown to be effective in reducing the expression of super-enhancer associated genes and inhibiting the growth of multiple cancers such as small cell lung cancer [15], MYCN-driven neuroblastoma cells [16], and triple-negative breast cancer [17]. These studies indicated the enormous potential for targeting transcriptional addiction in aggressive and therapeutically recalcitrant tumors. However, the mechanisms involved in CDK7-mediated tumor immune evasion are unclear in NSCLC.

MYC is dysregulated in more than half of human cancers and is usually in association with aggressive phenotype [18, 19]. MYC enhances the oncogenic transcriptional amplification program in cancers and plays a critical role in a variety of tumor biology including immune evasion, energy metabolism, invasion, angiogenesis, and proliferation [20]. It has been reported that MYC is amplified in NSCLC, and its expression is often upregulated in NSCLC patients with poorer outcomes [21]. Dysregulated MYC cooperates with HIF-1 to regulate the Warburg effect by induction of hexokinase 2 (HK2) [22]. MYC promotes immunity evasion by enhancing PD-L1 expression while MYC inactivation synergized with immune checkpoint blockades (ICBs) [23, 24]. Topper et al. utilize combined epigenetic therapy to reverse immune evasion and enable effective treatment of lung cancer via MYC depletion [25].

We hypothesized that CDK7 could regulate PD-L1 expression in an MYC-dependent manner. Herein, we analyzed transcriptomic data to identify that high CDK7 mRNA was associated with clinical outcomes in NSCLC by the GEPIA tool [26] and validated high CDK7 protein level as a prognostic factor by immunohistochemistry analysis in NSCLC patients’ tissue microarrays (TMAs) of two cohorts. Furthermore, we evaluated the effects of CDK7 inhibition in NSCLC cell lines by using the specific CDK7 inhibitor THZ1 and RNA silencing. Additionally, we showed that CDK7 inhibition reactivated immunity by suppressing p38α/MYC/PD-L1 and sensitized cancer cells to anti-PD1 therapy in vivo, which indicated CDK7 as an attractive target for epigenetic therapy of NSCLC. Last, CDK7/MYC/PD-L1 signature and infiltrating T cell status were collectively used to stratify NSCLC patients into different risk groups.

## Materials and methods

### Database analysis

The Cancer Genome Atlas (TCGA, https://www.cancer.gov/tcga) NSCLC data was analyzed to identify associations between mRNA expression of TFIIH subunits and clinical outcomes by the GEPIA (http://gepia.cancer-pku.cn/). GSE37745 [27] was re-analyzed to identify the association between CDK7 and survival outcomes. TCGA NSCLC data, GSE37745, The Cancer Cell Line Encyclopedia (CCLE, https://portals.broadinstitute.org/ccle) NSCLC cell line data, and The Cancer Proteome Atlas (TCPA, http://tcpaportal.org) NSCLC data were used for validation of correlation between CDK7/p38α/MYC axis and PD-L1.

### Patients and tissue microarrays

#### TMA cohort I

A lung cancer tissue microarray was obtained from Shanghai Outdo Biotech (#HLugA180Su05, Shanghai, China), which contained 94 carcinoma tissue and paired adjacent tissue. All patients had been pathologically diagnosed with lung adenocarcinoma.

#### TMA cohort II

The consecutive TMA cohort consists of consecutive NSCLC patients diagnosed at the Tongji Hospital during 2012–2014. Our study was conducted in accordance with US Common Rule, and archived primary NSCLC paraffin-embedded specimens were collected under a Human Research Ethics Committees protocol at Tongji Hospital with patients’ written formal consent. These patients have been followed over time. In this study, a total number of 231 lung adenocarcinoma patients were censored in 2018 with a median age of 57 years (range 27–81 years) at the time of diagnosis. The median follow-up was 707 days (range 0–2204 days). Specimens that are not suitable for evaluation in TMA are excluded due to missing or overlap of tissue cores.

### Immunohistochemistry

IHC analysis was performed on formalin-fixed paraffin-embedded tissue sections. The sections were deparaffinized, rehydrated, and stained with primary antibodies overnight at 4 °C. These antibodies were detected with biotinylated secondary antibody, followed by incubation with horseradish peroxidase-conjugated streptavidin-biotin complex. Finally, the sections were developed in diaminobenzidine and visualized under a light microscope or scanned by the NanoZoomer S360 Slide Scanner (Hamamatsu Photonics, Japan). The following primary antibodies were used: CDK7 (IHC, 1:100, CST #2916), p38α (IHC, 1:100, ABclonal #A14401), MYC (IHC, 1:100, ABclonal #A11029), PD-L1 (IHC, 1:100, CST #13684), Ki67 (IHC, 1:100, CST #9449), cleaved Caspase-3 (IHC, 1:500, CST #9661), CD8A (IHC, 1:100, Abcam #ab217344).

**Fig. 1:**
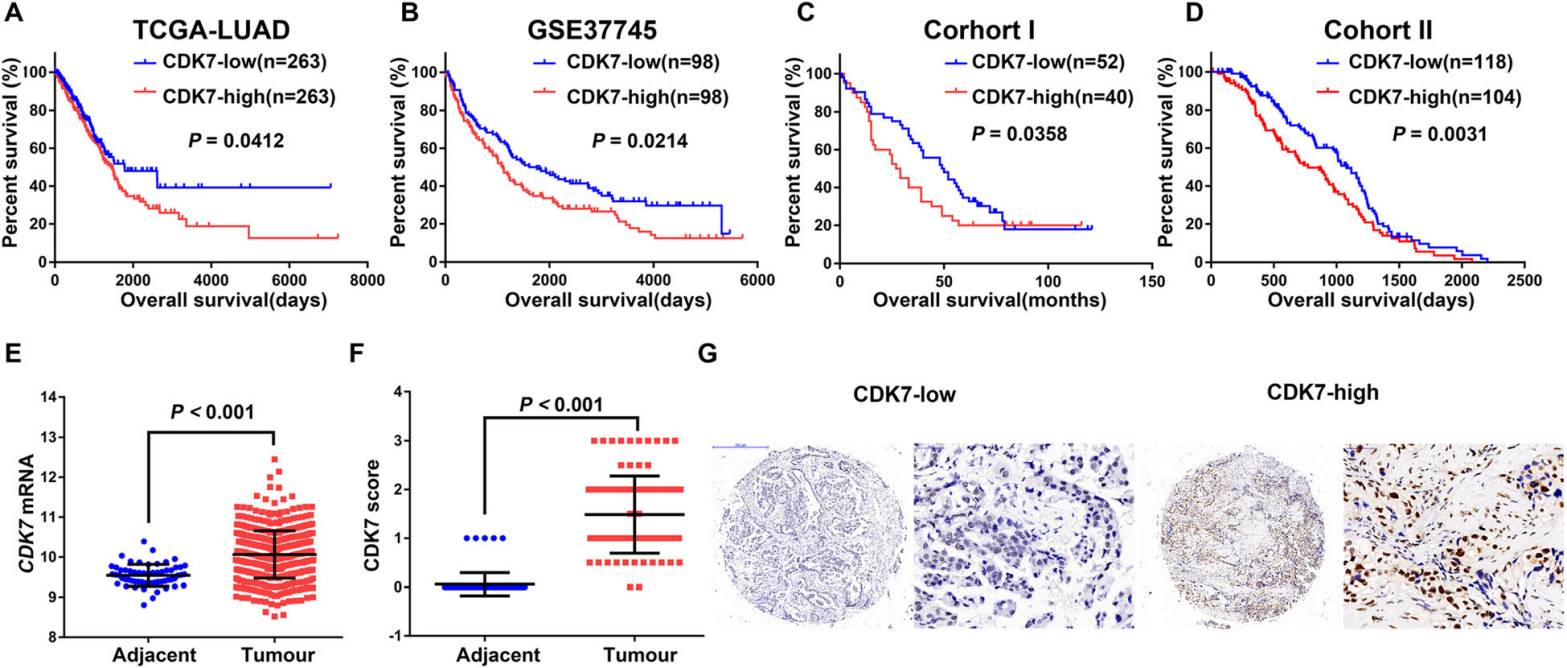
High CDK7 expression is associated with poor prognosis in NSCLC. **a** Kaplan-Meier survival curves (K-M curve) showing the relationship between *CDK7* mRNA level and OS in the TCGA LUAD data by GraphPad Prism Software (*n* = 526) (*P* = 0.0412). **b** K-M curve showing the relationship between *CDK7* mRNA level and OS in GSE37745 data (*n* = 196) (*P* = 0.0214). **c** K-M curve showing the relationship between *CDK7* protein level and OS in cohort I from Shanghai Outdo Biotech (*n* = 92) (*P* = 0.0358). **d** K-M curve showing the relationship between *CDK7* protein level and OS in cohort II from Tongji Hospital (*n* = 222) (*P* = 0.0031). **e** Data mining showing differential *CDK7* mRNA levels in adjacent and tumor tissue in TCGA LUAD data (*P* < 0.001). **f** The *CDK7* protein level in adjacent and tumor tissue in cohort I, as examined by immunohistochemistry (IHC) (*P* < 0.001). **g** Representative scanned images of tissue cores with low or high CDK7 by IHC. Left, original magnification, × 6; scale bar, 500 μm. Right, original magnification, × 400; scale bar, 50 μm

CDK7 staining was semi-quantified using IHC signal intensity in the tumor cell nuclei, scored as 0 to 3+ (no staining as 0, weak as 1, moderate as 2, and strong as 3). For p38α, MYC, and PD-L1, we calculated the score of each sample by multiplying the staining intensity with the percentage scale. The percentage of cells stained was categorized as follows: no positive cells as 0, less than 25% positive cells as 1, 25–50% positive cells as 2, 50–75% positive cells as 3, and more than 75% positive cells as 4. We divided the samples into the “high expression” group and the “low expression” by high expression criteria as CDK7 score ≥ 2, p38α score ≥ 2, MYC score ≥ 1, and PD-L1 score ≥ 1, respectively. Representative scanned images of tissue cores with low or high CDK7, p38α, MYC, and PD-L1 protein expression are shown in Fig. 1g and S7F-H.

### Evaluation of tumor-infiltrating lymphocytes

For the evaluation of tumor-infiltrating lymphocytes (TILs) score, we used semi-quantification to assess the TILs status according to the study [28] with some modifications. The scoring of TILs in TMA cohorts was performed in the same tissue cores used in IHC analysis by immunofluorescence (IF) staining of T lymphocytes (CD3, IF, 1:100, Abcam #ab16669), cytotoxic T cells (CD8, 1:100, IF, Santa Cruz Biotechnology #sc-7970), and Nuclei (DAPI). Based on the visual estimation of the proportion of CD3+ or CD8+ cell lymphocytes, TIL status was classified into 7 groups: ≤5%, 6~10%, 11~15%, 16~20%, 21~25%, 26~30%, >30%. By testing different cutoff values, we found that the number of low TIL patients (*n* = 87) is much closer to that of high TIL patients (*n* = 136) when 10% was chosen as the cutoff value. When combining different risk factors to predict survival outcomes, TIL status was classified into low TIL scores (≤ 10% TILs in tumor tissue) and high TIL scores (> 10% TILs in tumor tissue) in this study. The whole-tissue sections of morphologically normal human tonsil were included in each staining batch as positive control and to assess the interexperimental reproducibility. Representative scanned images of tissue cores with high or low TIL scores are shown in Figure S7I.

### RNA-seq and gene enrichment analysis

Gene expression analysis was conducted by RNA-seq for the conditions described in the relevant figures. Treated cells were harvested for RNA extraction using TRIzol. Reagent genomic and DNA was removed using DNase I (Takara). The sequencing library was constructed after high-quality RNA was quantified and then sequenced with the Illumina HiSeq X Ten (2 × 150 bp read length). The raw paired end reads were trimmed and quality controlled by SeqPrep (https://github.com/jstjohn/SeqPrep) and Sickle (https://github.com/najoshi/sickle) with default parameters. Then, clean reads were aligned separately to the reference genome. To identify differential expression genes between two different samples, the expression level of each transcript was calculated according to the fragments per kilobase of exon per million mapped reads method. RSEM (http://deweylab.biostat.wisc.edu/rsem/) was used to quantify gene abundances. The R statistical package software EdgeR (http://www.bioconductor.org/packages/2.12/bioc/html/edgeR.html) was utilized for differential expression analysis.

Differential expression genes (DEGs) were defined as |fold change| ≥ 2 and *P* value ≤ 0.05 in transcription for drug-treated conditions over mock for each sample studied. In addition, functional-enrichment analysis including KEGG pathways, Gene Ontology (GO) enrichment [29], and gene set enrichment analysis (GSEA) [30] were performed. Only categories that were below the DAVID *P* value of 0.05 and containing at least 5 genes per pathway are reported.

### Animal experiments

Mice were purchased from Nanjing Biomedical Research Institute of Nanjing University, China, and housed under pathogen-free conditions. All studies were performed following the NIH Guidelines for the Care and Use of Laboratory Animals and approved by the Animal Care and Use Committee of Huazhong University of Science and Technology. Three murine models were used in this study and established as follows:

(1) Balb/c nu/nu mice (5 weeks old, female) were subcutaneously (s.c.) injected with 5 × 10^6^ H460 or H292 cells on their dorsal flanks.

(2) A patient-derived xenograft (PDX) mouse model was derived from a patient with lung adenocarcinoma. We first transplanted the tumor tissue (directly obtained from a patient with lung adenocarcinoma) into a Balb/c nu/nu mouse. When the patient-derived xenograft (PDX) reaches more than three times in volume in the donor mouse, the tissue was collected and sectioned into 2 × 2 × 2 mm^3^ fragments. These fragments were then implanted subcutaneously in the flank of Balb/c nu/nu mice (5 weeks old).

(3) C57BL/6 mice (5 weeks old, female) were s.c. injected with 5 × 10^5^ Lewis lung carcinoma (LLC) cells or B16-F10 melanoma, both of which are syngeneic to C57BL/6, on their dorsal flanks. Treatment was initiated when palpable tumors in each group achieved 5–9 mm in diameter. Mice were randomized such that different groups had similar average tumor volumes before treatment initiation. THZ1 was administered intraperitoneally (i.p.) (10 mg/kg, twice daily) [15], and LY2228820 was delivered by oral gavage (15 mg/kg daily) [31]. AntiPD-1 antibody was injected (i.p.) (10 mg/kg twice weekly) [25]. CD8^+^ T cells were depleted in a subset of mice by intraperitoneal injection of CD8a depletion antibody (BioXcell, 2.43 clone), 3 times per week at 150 μg per animal. Drugs were given for the duration of the study, and comparable control treated with vehicle or rat IgG2a isotype antibody was run in parallel. Each animal was tracked individually for tumor growth by external caliper measurements of subcutaneous protruding tumors, and an approximate tumor volume was calculated using the formula: length × width^2^ × 0.5. Animals were also weighed 3 times a week. At the end of experiments, mice were sacrificed, and tumors were harvested, weighed, and prepared for analysis.

### Fast drugs sensitivity assay

To determine the combination of THZ1 and LY2228820 in patient-derived tumor cells, PDX tumors were digested with tissue dissociation buffer (0.1% collagenase, 0.01% hyaluronidase, 0.01% DNase I in Hank’s Balanced Salt Solution) for 1 h at room temperature (RT) to obtain small fragment suspension. The small PDX tissue was seeded in 96 well plates coated with matrigel matrix and treated with different regimens. After 3 days, the cell viability was determined by CCK8 assay and analyzed using Graphpad Prism.

### Flow cytometry profiling tumor-infiltrating lymphocytes

Primary tumors were harvested from treated mice at the end of experiments. Tissue was then digested using a mixture of collagenase, hyaluronidase, and DNase [32]. The resulting single-cell suspension was counted and plated in complete media with or without an eBiosciences stimulation cocktail (1:1000) for 4 h at 37 °C. For cell surface staining, cells were kept at 4 °C and stained with APC/Cy7-conjugated antiCD45 (1:100, Biolegend #103116), APC-conjugated antiCD8a (1:100, Biolegend #100712), and APC-conjugated antiPDL1 (1:100, Biolegend #124308). For intracellular staining, the eBioscience™ Intracellular Fixation & Permeabilization Buffer Set (Invitrogen™) was used, and cells were stained with BV-421-conjugated antiIFN-γ (1:100, Biolegend #505830). Data were acquired on a CytoFLEX (Beckman Coulter) and analyzed with the FlowJo software. The gating strategy is outlined [33], and representative FACS images are shown in Figure S6H-I. After gating by SSC and FSC of all cells, then, lymphocytes were gated by CD45 and CD8. Next, the percentages of cell subtypes are quantified, and IFN-γ levels of CD8^+^CD45^+^ are measured. Both CD45 and CD8 negative of all cells are gated as tumor cells and measured by PD-L1 mean fluorescence intensity (MFI).

Detailed methods of Western blot, RT-PCR, gene knockdown or overexpression, cell viability assay, cell growth assay, colony formation assay, caspase-3 activity assay, TUNEL assays, quantitative interferon (IFN)-γ, flow cytometry analysis of apoptosis, MYC transcriptional activity, extracellular flux analysis via seahorse metabolic system, lactate and NAPDH/NAPD+ measurements, and reactive oxygen species (ROS) detection are in Supplementary materials or described previously [34–37].

### Statistical analysis

All the data came from at least three independent experiments and were shown in the form of mean ± standard deviation (SD) unless otherwise stated. Unpaired two-tailed Student’s test was used to compare two independent groups. One-way analysis of variance (ANOVA) was used when three or more independent groups were compared. For survival analysis, the expression of indicated genes was roughly treated as a binary variant and divided into “high” and “low” level. Then data were plotted and compared using the log-rank test or Gehan-Breslow-Wilcoxon test. The CompuSyn method was used to assess the synergy of drug combinations [38]. A combination index (CI) value of less than 0.9 was significant. Pearson’s correlation analysis was used to assess the correlation between two genes. No statistical method was used to predetermine the sample size. A two-tailed test with *P* < 0.05 was significant. The analysis was performed with the GraphPad Prism Software version 7.

## Results

### CDK7 is highly expressed in NSCLC tumor tissue and is a poor prognostic predictor in NSCLC

Gene expression is under tight control for physiological cell homeostasis. However, it is frequently dysregulated in cancer. The general transcription factor TFIIH is an integral component of the RNA polymerase II pre-initiation complex, and it plays a pivotal role in transcriptional regulation [14]. After analysis of the 10-subunit TFIIH complex in TCGA NSCLC dataset by using GEPIA tool, high *CDK7* mRNA was identified to be specifically associated with poor prognosis. When a median *CDK7* mRNA expression cutoff point was used for stratification, high *CDK7* mRNA expression was correlated with both reduced disease-free survival (DFS) and overall survival (OS) (Fig. 1a and S1A). Further survival analysis was carried out in GSE37745 data (*n* = 196), which suggested that *CDK7* mRNA could predict OS outcomes in NSCLC (Fig. 1b). To further validate our findings, we examined *CDK7* protein expression by immunohistochemistry (IHC) in two independent cohorts of NSCLC patients. In both cohort I (*n* = 92) and cohort II (*n* = 222), high *CDK7* protein expression was significantly associated with poorer OS in NSCLC patients (Fig. 1c, d). Compared with adjacent tissue, *CDK7* mRNA and protein levels in tumors were higher (Fig. 1e–g). Data mining of CCLE also revealed that *CDK7* mRNA was higher in NSCLC cell lines than that in SCLC cells (Figure S1B-C). These findings indicate that CDK7 could be a prognostic factor and a promising therapeutic target in NSCLC.

### Targeting CDK7 promotes apoptosis and suppresses NSCLC growth in vitro and in vivo

**Fig. 2:**
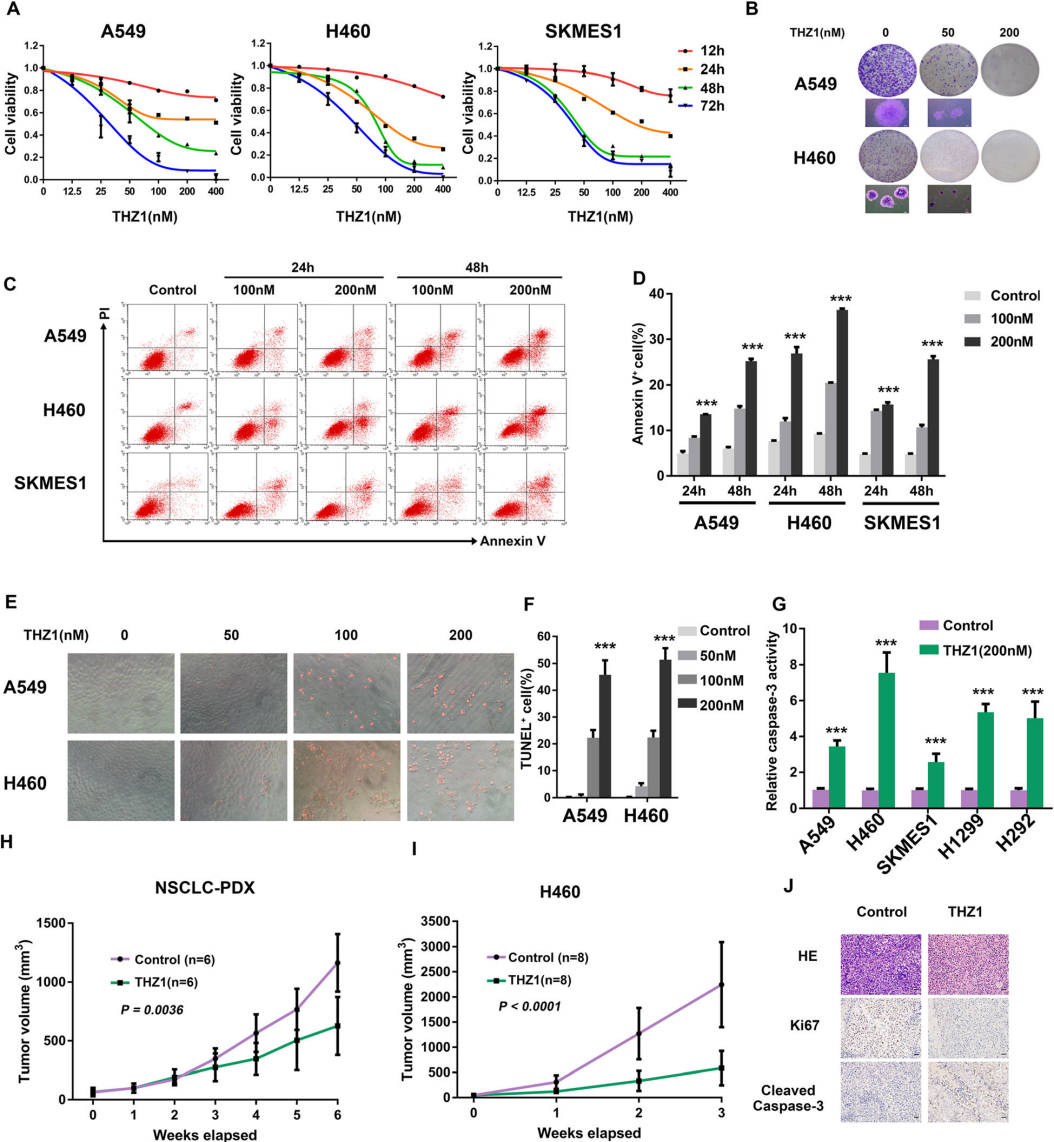
CDK7 inhibitor THZ1 promotes apoptosis and suppresses NSCLC growth in vitro and in vivo. a CCK8 assay depicting the changes in the viability of A549, H460, and SKMES1 cells treated with different doses of THZ1 for time points as indicated (*n* = 3). b Representative images showing colony formation of A549 and H460 cells treated with vehicle or THZ1. c Representative images of annexin V/propidium iodide staining showing increased apoptosis in NSCLC cells treated with THZ1. d Quantification of the annexin V positive cell ratio (*n* = 3) (***P < 0.001 as compared to vehicle group). e Representative images showing increased TUNEL positive cells (with nucleus stained in red) after THZ1 treatment at 48 h (× 40 magnification). f Quantification of the TUNEL positive cell ratio (*n* = 3) (***P < 0.001 as compared to vehicle group). g Caspase-3 activity was measured after NSCLC cells were treated with THZ1 (200 nM) for 48 h. Results are presented as fold-increase to vehicle-treated samples (*n* = 3) (*** *P* < 0.001). h Tumor growth curves of a lung adenocarcinoma PDX model treated with either vehicle or THZ1. Data represent mean ± SD (*n* = 6) (*P* = 0.0036). i Tumor growth curves of the H460 xenograft model treated with either vehicle or THZ1. Data represent mean ± SD (*n* = 8) (*P* < 0.0001). j H&E and IHC staining of Ki67 and cleaved caspase-3 in tumor tissue sections from H460 xenograft. Original magnification, × 400; scale bar, 20 μm

To determine the effects of CDK7 on NSCLC, we silenced CDK7 (Figure S2A) and showed that NSCLC cell lines with ablated CDK7 demonstrated significantly decreased proliferation (Figure S2B). Recently a covalent CDK7 inhibitor THZ1 has been reported as a promising drug for different types of cancers such as small cell lung cancer (SCLC) by preferentially targeting super-enhancer-driven transcription factor genes including *MYC* family proto-oncogenes and neuroendocrine lineage-specific factors [15]. Therefore, THZ1 could be a potent drug for NSCLC and was used to investigate the functional role of CDK7 in NSCLC. First, THZ1 inhibited NSCLC viability in a time- and dose-dependent manner (Fig. 2a and S2C). Moreover, THZ1 treatment resulted in less and smaller colonies of NSCLC cell lines in a relatively long-time course (Fig. 2b and S2D). To explain the mechanisms by which THZ1 reduced viability of NSCLC cells, we assessed apoptotic cell death caused by CDK7 inhibition. In annexin V/PI staining and TUNEL assay, THZ1-treated NSCLC cells showed significantly the increased ratio of annexin V positive (Fig. 2c, d) and TUNEL positive cells (Fig. 2e, f) respectively, which showed induction of apoptosis. Additionally, both CDK7 silencing and CDK7 inhibitor THZ1 upregulated caspase-3 enzyme activity in NSCLC (Fig. 2g and S2E). We subsequently performed cell cycle analysis and observed G2/M phase arrest upon THZ1 treatment in A549 cells, but no significant changes in H460 cells (Figure S2F-G). Next, we tested the antitumor effects of THZ1 in xenograft models established from H460 cells and in a lung adenocarcinoma PDX model. As expected, THZ1 suppressed NSCLC tumor growth and tumor weights in vivo (Fig. 2h and i and S2H-J). Importantly, no significant loss of body weight (Figure S2K-L) or other common toxic effects were observed. Furthermore, IHC analysis of ki67 and cleaved caspase-3 staining confirmed the dramatic decrease of cell proliferation and the increase of apoptotic cancer cells in the H460 xenograft upon THZ1 administration (Fig. 2j, S2M-N). Collectively, these data demonstrated that CDK7 inhibition can suppress NSCLC growth via induction of apoptosis in vitro and in vivo.

### THZ1 suppresses p38α/MYC signaling in NSCLC

**Fig. 3:**
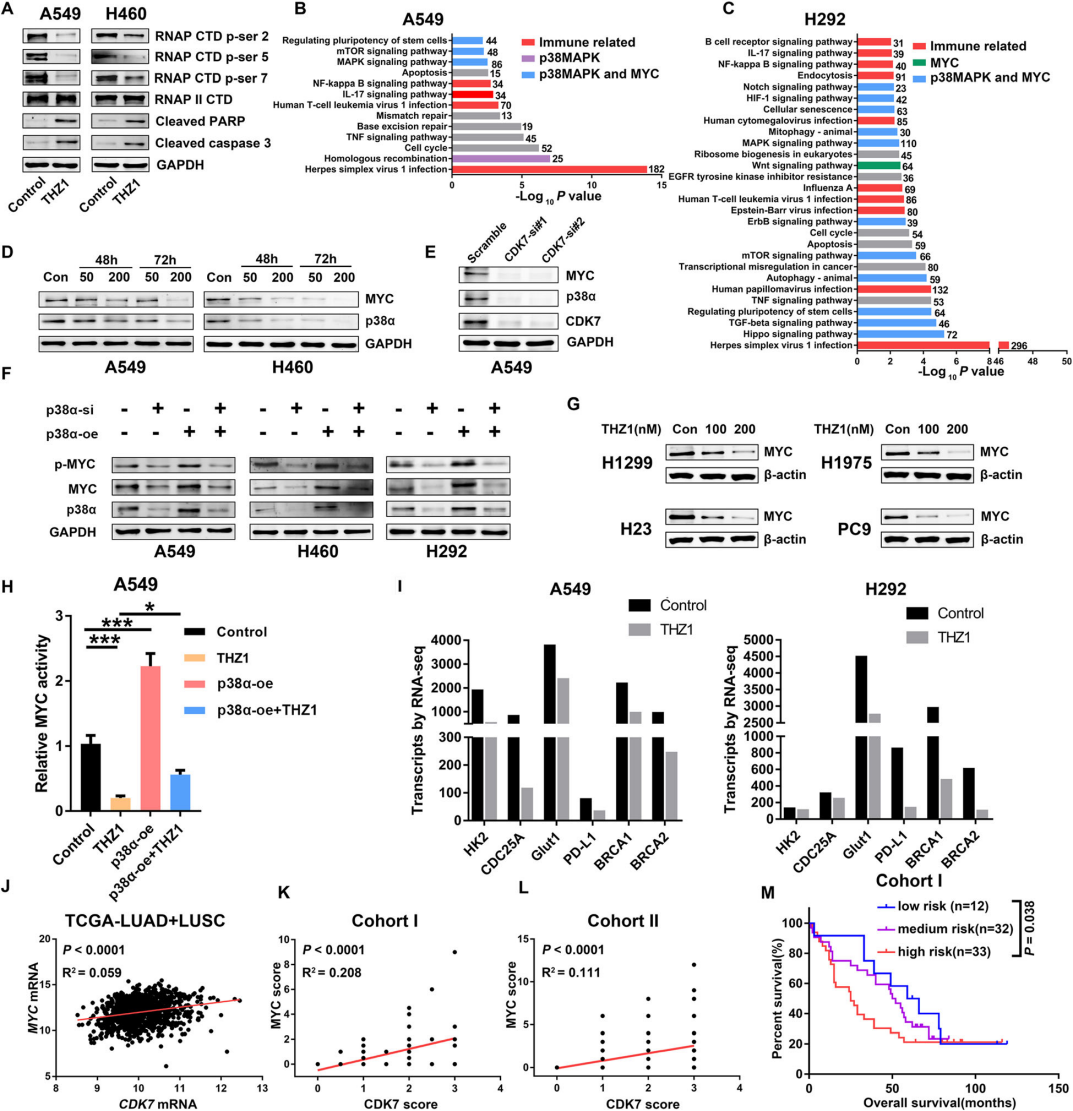
CDK7 regulates the p38α/MYC pathway in NSCLC. a Changes of RNAPII C-terminal domain (CTD) phosphorylation and apoptosis markers in A549 and H460 cells after vehicle or THZ1 treatment at 48 h. b KEGG pathway analysis of differential expression genes for THZ1(200 nM, 24 h) treated cells over the vehicle in A549 cells. **c** KEGG pathway analysis of differential expression genes for THZ1 (200 nM, 24 h) treated cells over the vehicle in H292 cells. **d** Immunoblot analysis of p38α and MYC in A549 (left) and H460 (right) cells treated with vehicle or THZ1 at indicated time points. GAPDH was used as a loading control. **e** Immunoblot analysis of p38α, MYC, and CDK7 in A549 cells transfected with either scramble or CDK7 siRNAs. GAPDH was used as a loading control. **f** Immunoblot of MYC proteins after NSCLC cells transfected with p38α siRNAs or p38α vector. GAPDH was used as a loading control. **g** Immunoblot of MYC proteins in H1299, H1975, H23, and PC9 cells after THZ1 treatment with indicated dose at 48 h. **h** Luciferase reporter assays were performed to evaluate the transactivation potential of MYC in cells treated with THZ1 or transfected with p38α vector (*n* = 3) (*P < 0.05; ***P < 0.001). **i** Changes of MYC-targeted genes after THZ1 treatment in A549 and H292 cells by RNA-seq. **j** Correlation between *CDK7* mRNA level and *MYC* mRNA level in TCGA NSCLC data (*P* < 0.0001). **k** Correlation between *CDK7* protein level and *MYC* protein level in cohort I samples (*P* < 0.0001). **l** Correlation between *CDK7* protein level and *MYC* protein level in cohort II samples (*P* < 0.0001). **m** Kaplan-Meier survival analysis of patients with different risk scores. High expression levels of CDK7, p38α, and MYC were defined as three risk factors. Patients were stratified into three risk groups with different survival outcomes as follows: low-risk group = none of the three were high expression (*n* = 12); medium-risk group = only one of the three was high expression (*n* = 32); high-risk group = at least two of the three were high expression (*n* = 33) (*P* = 0.038)

CDK7, as a core component of TFIIH, regulates transcriptional process via phosphorylating the serine 2, 5, and 7 of the RNAPII C-terminal domain (RNAPII CTD) [7, 39]. Consistently, we observed decreased phosphorylation S2, S5, and S7 of RNAPII in both A549 and H460 cells at 48 h after THZ1 treatment, which indicated effective inhibition of global transcription, concomitant with the appearance of apoptotic markers including cleaved caspase-3 and cleaved PARP (Fig. 3a). Thus, disruption of RNAPII transcription through CDK7 inhibition appears to potently impair NSCLC cell viability. To understand the mechanisms underlying the cytotoxic effects of THZ1 and investigate THZ1-induced transcriptional effects on NSCLC cells, we next performed RNA-seq in A549 and H292 cells after vehicle or THZ1 treatment (200 nM, 24 h). Notably, THZ1 treatment led to a widespread transcriptional reduction in mRNA levels of actively transcribed genes (Figure S3A-B). Clustering of the differentially expressed genes (DEGs) via GO analysis showed that THZ1-regulated genes were mainly involved in the transcription process (Figure S3C-D). Further, KEGG pathway analysis revealed that THZ1-regulated pathways were enriched in three subsystems [25]: immune-related pathways, p38α (encoded by *MAPK14*)-related pathways, and MYC-related pathways (Fig. 3b, c). Consistent with the results that THZ1 induced apoptosis and growth arrest in NSCLC, the enriched pathways included the apoptosis pathway. Besides, researches have shown that p38α could regulate MYC protein level via AP1-REGγ-Wnt/β-catenin pathway [40] and stabilize MYC mRNA via tristetraprolin (TTP) phosphorylation [41]. Interestingly, both p38α [42] and MYC [24, 25] are reported to be linked with the remodeling of tumor immune microenvironment, suggesting that CDK7/p38α/MYC pathway may play a prominent role in the tumor immune microenvironment of NSCLC.

First, we found that THZ1 downregulated both p38α and MYC protein levels in a dose- and time-dependent manner in A549 and H460 cells (Fig. 3d). CDK7 knockdown resulted in decreased p38α and MYC protein levels in A549 cells (Fig. 3e). THZ1 and CDK7 silencing downregulated p38α and MYC mRNA levels (Figure S3E-F). Furthermore, p38α knockdown dramatically decreased MYC protein level in different NSCLC cell lines, and overexpression of p38α led to the accumulation of MYC protein (Fig. 3f). However, p38α overexpression could only partially rescue THZ1-induced MYC ablation (Figure S3G), suggesting that THZ1 might decrease MYC protein level via multiple mechanisms. Besides, we found that CDK7 inhibition downregulated MYC protein levels in H1299, H1975, H23, and PC9 cells (Fig. 3g). Next, we verified that THZ1 could abolish phosphorylated MYC, which is crucial for the transcriptional activity of MYC, in a time-dependent way in H292 cells (Figure S3H). As shown in Fig. 3h, we found that THZ1 treatment suppressed MYC transcriptional activity, and p38α overexpression could partially rescue this effect of THZ1. We also determined MYC mRNA stability in A549 cells and found p38α knockdown and p38α inhibitor LY2228820 (Ralimetinib) decreased MYC mRNA stability (Figure S3I). RNA-seq data also showed that THZ1 could diminish transcripts of MYC-targeted genes, such as HK2, CDC25A, GLUT1 (SLC2A1), PD-L1 (CD274), BRCA1, and BRCA2 (Fig. 3i), which are important mediators of MYC’s function in tumor metabolism, immune evasion, and DNA repair. Then we validated the RNA-seq data by RT-PCR and showed that THZ1 downregulated p38α and MYC associated genes (Figure S3J).

Then, the analysis of data extracted from the TCGA-TCPA NSCLC database and GSE37745 demonstrated a strong positive correlation among the CDK7/p38α/MYC axis (Fig. 3j, S3K-L). Similarly, we observed that the CDK7 protein level correlated with MYC protein expression in cohort I (Fig. 3k) and cohort II (Fig. 3l). To investigate the prognostic values of the CDK7/p38α/MYC pathway, we used different proteins in combination to predict the survival outcome of NSCLC patients. Although p38α or MYC protein level alone did not differentiate NSCLC patients with good versus poor clinical outcome in cohort I (Figure S3M and S3O), patients with CDK7/p38α double high expression had a shorter OS than the others (Figure S3N) while patients with CDK7/MYC double low expression had better OS outcomes than the others (Figure S3P). Considering CDK7, p38α, and MYC as three risk factors, we could stratify patients into three risk groups with different clinical outcomes (Fig. 3m). These results support that the CDK7/p38α/MYC pathway is a crucial survival factor in NSCLC and may relate to eventual clinical efficacy.

### The combination of THZ1 and p38α inhibitor synergistically suppressed NSCLC by downregulating MYC

**Fig. 4:**
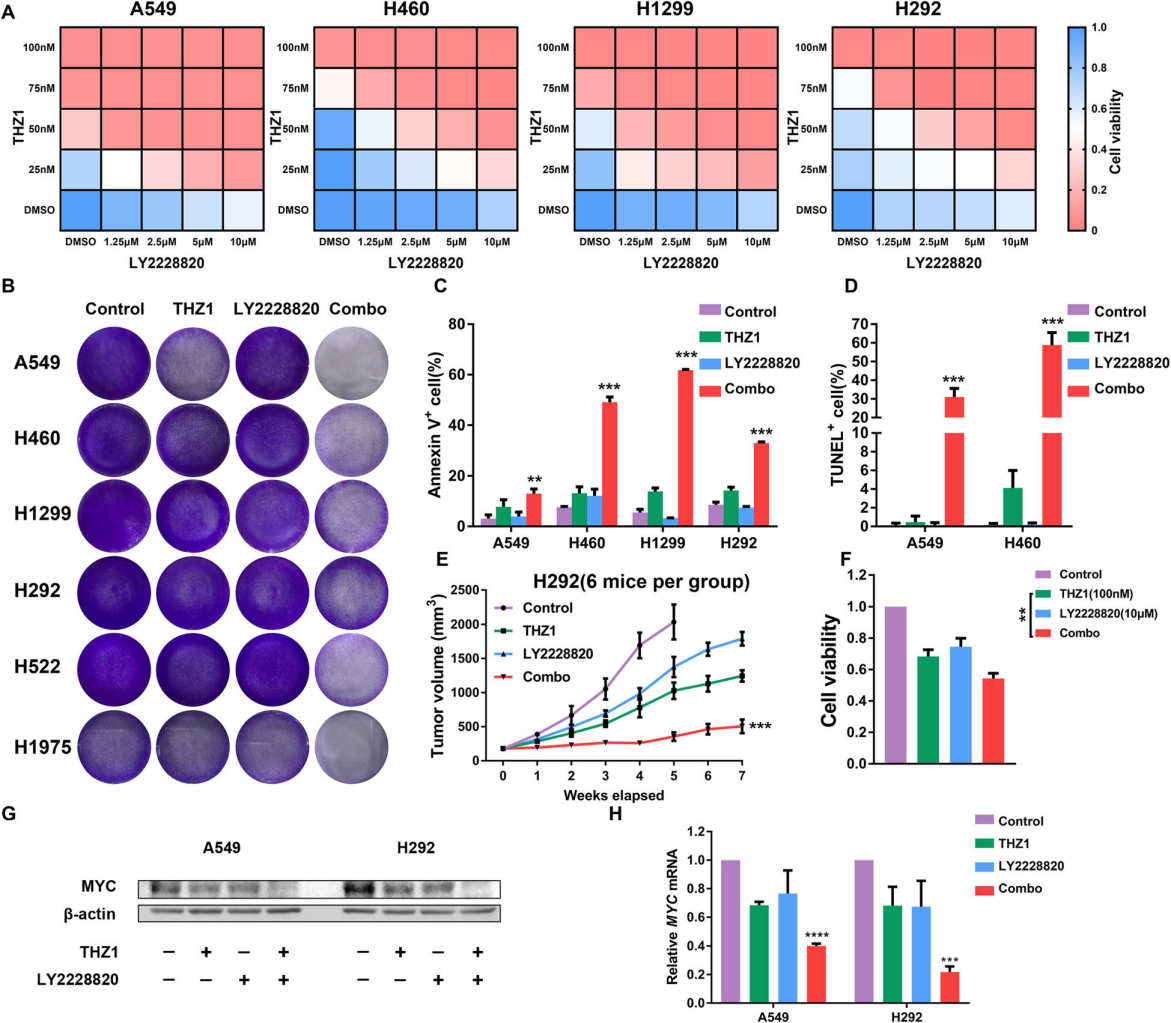
CDK7 inhibition sensitizes cancer cells to p38α inhibitor in NSCLC. **a** CCK8 viability assay following escalating concentrations of THZ1 and LY2228820 treatment for 48 h in NSCLC cells. **b** Representative images showing the response of NSCLC cell lines to the combination of THZ1 and LY2228820 treatment at 96 h. Cells were treated with THZ1 (50 nM), LY2228820 (5 μM), or the combination for 48 h and then cultured with fresh medium for another 48 h. In the end, cells were stained with crystal violet solution and photographed. **c** Quantification of the annexin V positive cell ratio after the combination of THZ1 (50 nM) and LY2228820 (5 μM) for 48 h (*n* = 3) (**P < 0.01; ***P < 0.001 as compared to THZ1 group). **d** Quantification of the TUNEL positive cell ratio after the combination of THZ1 (50 nM) and LY2228820 (5 μM) for 48 h (*n* = 3) (***P < 0.001 as compared to THZ1 group). **e** Tumor growth curves of the H292 xenograft model treated with the combination of THZ1 and LY2228820 (*n* = 6) (***P < 0.001 as compared to THZ1 group). **f** CCK8 cell viability assay following THZ1 (100 nM) and LY2228820 (10 μM) treatment for 72 h in cancer cells from the PDX model (*n* = 4) (**P < 0.01). **g** Changes of MYC protein levels in A549 and H292 cells after the combination of THZ1 (50 nM) and LY2228820 (5 μM) for 48 h. β-actin was used as a loading control. **h** Relative changes of MYC mRNA levels in A549 and H292 cells after the combination of THZ1 (50 nM) and LY2228820 (5 μM) for 48 h. Results are normalized to β-actin (*n* = 3) (***P < 0.001; ****P < 0.0001 as compared to control group)

In response to stress, p38α is activated in cancer cells by phosphorylating different substrates and regulating cytokine production in the tumor microenvironment, indicating its crucial role in tumor growth, metastasis, drug resistance, and reprogramming tumor immune microenvironment. Therefore, we hypothesized that inhibition of CDK7 and p38α could enhance antitumor effects in combination. To test this hypothesis, we combined THZ1 treatment with the p38α inhibitor LY2228820 in four NSCLC cell lines. As is evident from the heatmap representation of the CCK8 cell viability assay, the combination treatment caused a synergistic inhibition of cancer cell proliferation (Fig. 4a) with all mean combination index (CI) < 0.8 (Figure S4A). The synergistic effects of THZ1 and LY2228820 were also confirmed in cell growth assay in six NSCLC cell lines for 96 h (Fig. 4b). Next, we assessed the combination treatment using annexin V/PI staining and TUNEL assay and observed that THZ1 with LY2228820 treatment synergistically promoted apoptosis in NSCLC cells (Fig. 4c, d, S4B-C). We then found an enforced antitumor effect in the combined THZ1 and LY2228820 treatment group compared to each drug alone group in H292 xenograft models (Fig. 4e), without increased toxicity measured by body weight (Figure S4D). Moreover, in fast drug sensitivity assay, THZ1 and LY2228820 combination killed more lung adenocarcinoma cells from the PDX model than each drug alone (Fig. 4f). In addition, THZ1 and LY2228820 synergistically decreased the MYC protein and mRNA levels in A549 and H292 cells (Fig. 4g, h). Overall, our data provided an approach for therapy of NSCLC with THZ1 and LY2228820 in combination.

### THZ1 downregulates PD-L1 expression by inhibiting MYC activity in NSCLC

**Fig. 5:**
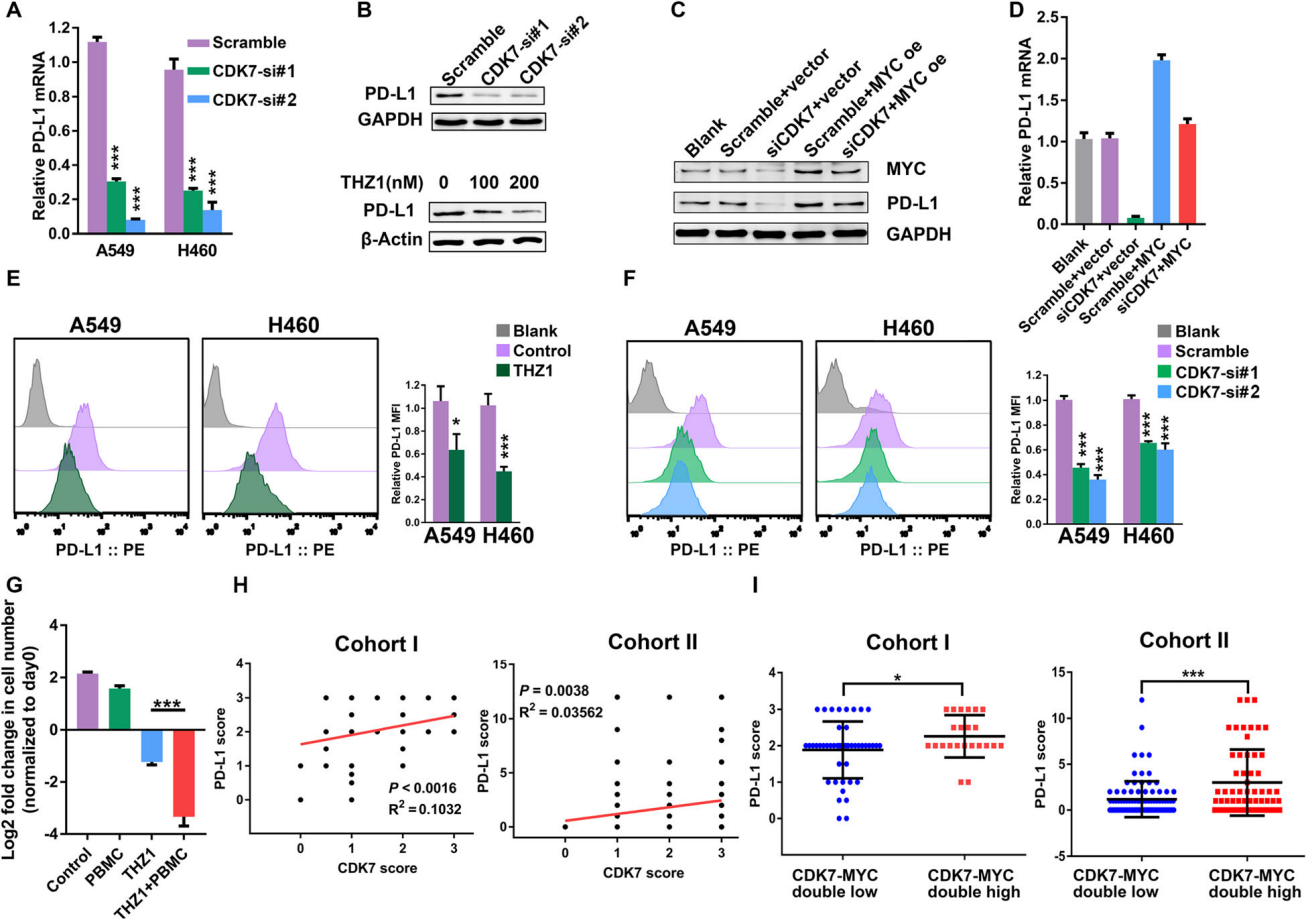
THZ1 downregulates PD-L1 expression by inhibiting MYC signaling. **a** Quantitation of relative PD-L1 mRNA expression in NSCLC cell lines transfected with either scramble or CDK7 siRNAs. Results are normalized to GAPDH (*n* = 3) (***P < 0.001 as compared to scramble group). **b** Changes of PD-L1 protein level in A549 cells transfected with either CDK7 siRNAs (compared to scramble group) or treated with THZ1 for 48 h (compared to vehicle group). GAPDH was used as a loading control. **c** Immunoblot of MYC and PD-L1 proteins after A549 cells transfected with CDK7 siRNAs, MYC vector, or the combination. GAPDH was used as a loading control. **d** Quantitation of relative PD-L1 mRNA level in A549 cells transfected with CDK7 siRNAs, MYC vector, or the combination. GAPDH was used as a loading control. **e** Representative images and quantitation of PD-L1 level on surfaces of NSCLC cell lines treated with THZ1 (200 nM) at 24 h (*n* = 3) (*P < 0.05; ***P < 0.001). **f** Representative images and quantitation of PD-L1 level on surfaces of NSCLC cell lines transfected with either scramble or CDK7 siRNAs (*n* = 3) (***P < 0.001 as compared to scramble group). **g** NSCLC cells were pretreated with vehicle or THZ1 (200 nM) and then cocultured with PBMC (1:10) for 3 days. Left y-axis indicates log 2 of fold change in cell number at 3 days relative to day 0 (*n* = 3) (***P < 0.001). **h** Correlation between PD-L1 protein level and CDK7 protein level in cohort I and cohort II tumor samples. **i** Patients with CDK7 and MYC double high levels had a higher PD-L1 score than patients with CDK7 and MYC double low based on cohort I data (*P < 0.05) and cohort II data (***P < 0.001)

Immune checkpoint blockades, such as antibodies targeting PD-1 and PD-L1, can induce robust and durable responses to different types of cancers, including NSCLC [43]. PD-L1 is a known transcriptional target of MYC, and MYC inactivation enhanced the antitumor immune response via downregulating CD47 and PD-L1 expression [24]. Thus, we speculated that CDK7 ablation could prevent the immune escape of tumor cells by suppressing the MYC/PD-L1 axis. First, CDK7 knockdown decreased PD-L1 mRNA and protein level (Fig. 5a, b). THZ1 treatment also downregulated the PD-L1 protein level (Fig. 5b). Then, MYC overexpression rescued CDK7 inhibition induced-PD-L1 decrease in both protein and mRNA levels (Fig. 5c, d), which matched with our hypothesis. Moreover, MYC overexpression also could reverse THZ1-induced PD-L1 downregulation (Figure S5A-C). Importantly, both THZ1 and CDK7 knockdown reduced the PD-L1 expression on NSCLC cell surfaces (Fig. 5e, f).

MYC is a master regulator of tumor energy metabolism via regulation of enzymes and transporters such as hexokinase (HK2) and glucose transporters (GLUT) (Fig. 3i). To test the hypothesis that CDK7 could modulate tumor glucose metabolism via suppressing MYC pathway, we found that THZ1 treatment (200 nM, 24 h) inhibited glycolysis via the extracellular acidification rate (ECAR) analysis and suppressed oxidative phosphorylation via oxygen consumption rate (OCR) analysis (Figure S5D) in seahorse metabolic analysis system. More specifically, analysis of ECAR and OCR data discovered that both glycolytic capacity and glycolytic reserve were prohibited upon THZ1 exposure in ECAR assay while ATP production, basal OCR, and maximal respiration were decreased in OCR assay. In addition, we showed that CDK7 inhibition suppressed extracellular lactate release (Figure S5E) and decreased NADPH/NADP+ ratio (Figure S5F). These metabolic disturbances could cause ROS upregulation in NSCLC (Figure S5G). Tumor-derived lactate has been reported to cause an immunosuppressive microenvironment through upregulating PD-L1 in human lung cancer cells [44]. As showed in Figure S5H, adding lactate increased PD-L1 on tumor surfaces and partial rescued THZ1-induced PD-L1 decrease, which showed that THZ1-induced extracellular lactate decline may lead to decreased PD-L1 expression.

Next, we assessed tumor proliferation after THZ1 treatment and cocultured with peripheral blood mononuclear cells (PBMC) [45]. THZ1 could kill more tumor cells in the presence of PBMC (Fig. 5g). Coculturing of PBMC with THZ1-pretreated H1975 cells (high PD-L1) [46] led to an increase of IFN-γ production compared with cocultured vehicle-treated H1975 cells. Consistently, adding Atezolizumab (anti-PD-L1 antibodies) also upregulated IFN-γ secretion in the PBMC-H1975 coculture system (Figure S5I). These data indicated the effect of THZ1-induced PD-L1 decrease on anticancer immunity. Data mining showed a tight positive correlation between PD-L1 mRNA level and CDK7-p38α-MYC axis in the TCGA-TCPA NSCLC data and GSE37745 (Figure S5J-K). Besides, patients with CDK7 and MYC double high expression had a higher PD-L1 mRNA level than patients with CDK7 and MYC double low expression based on TCGA-TCPA NSCLC data, GSE37745, and CCLE NSCLC cell line data (Figure S5L-N). Of note, we also found that the PD-L1 protein level correlated with the CDK7 protein level in cohort I and cohort II tumor samples (Fig. 5h). Additionally, patients with CDK7 and MYC double high expression had a higher PD-L1 protein level than those with CDK7 and MYC double low expression in both cohort I and cohort II (Fig. 5i). Finally, we proposed that THZ1 could suppress PD-L1 expression by inhibiting the MYC pathway and prohibiting extracellular lactate release in NSCLC.

### THZ1 enhances antiPD-1 therapy efficacy by recruiting CD8^+^ T cells in NSCLC

**Fig. 6:**
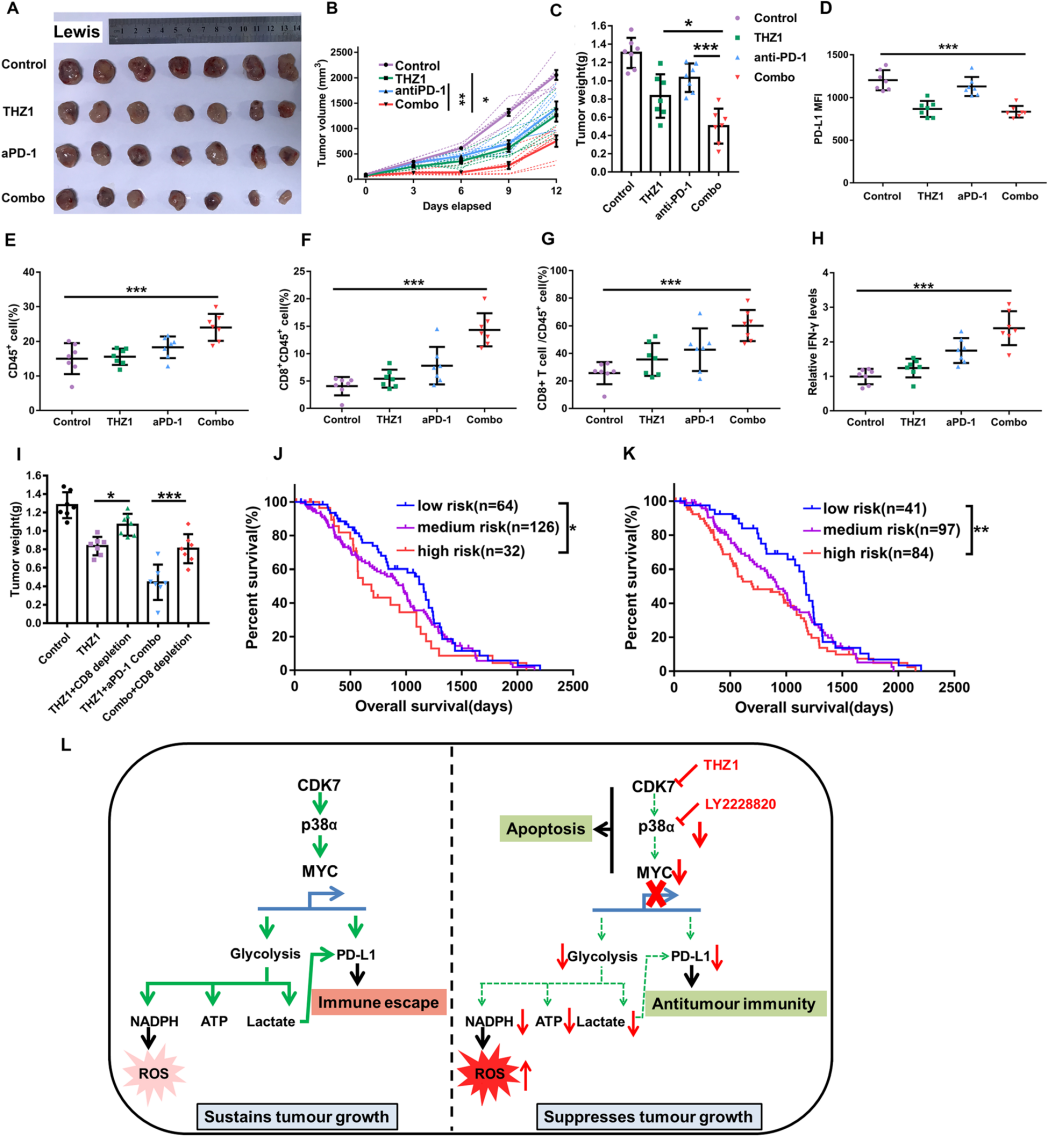
CDK7 inhibition stimulates antitumor immunity and sensitizes NSCLC to antiPD-1 therapy. **a** Photographs of tumors from the Lewis lung cancer model treated with the combination of THZ1 and antiPD-1 antibody (*n* = 7). **b** Tumor growth curves of mice from the Lewis lung cancer model. The dashed line represents a single mouse data in each group and the solid line represents the mean value in different groups. Error bars represent ± SEM (*P < 0.05; **P < 0.01). **c** Weights of tumors from mice in Lewis lung cancer model at the endpoint (*P < 0.05; ***P < 0.001). **d** Quantitation of PD-L1 level on tumor surfaces from the Lewis lung cancer model (***P < 0.001). **e** Quantification of percentages of CD45^+^ cells in tumors from the Lewis lung cancer model treated with the combination of THZ1 and antiPD-1 antibody (***P < 0.001). **f** Quantification of percentage of CD45^+^CD8^+^ cells in tumor from the Lewis lung cancer model treated with the combination of THZ1 and antiPD-1 antibody (*n* = 7) (**P < 0.01; ***P < 0.001). **g** The ratios of CD8^+^ T cell/CD45^+^ cell in tumors from the Lewis lung cancer model (***P < 0.001). **h** IFN-γ in the tumors from the Lewis lung cancer model was measured (***P < 0.001). The collected tumors were homogenized and detected by using Quantikine ELISA (R&D Systems). **i** C57BL/6 mice bearing Lewis tumor were treated with THZ1 or the combination of THZ1 and antiPD-1 with or without CD8^+^ T depletion by antibodies. The tumor burden of different groups was quantified by tumor weights at the endpoint (*P < 0.05; ***P < 0.001). **j** Kaplan-Meier survival analysis of patients with different risk scores by CDK7 protein level and tumor-infiltrating lymphocyte (TIL) scores in cohort II. High CDK7 protein level and low TIL scores were defined as two risk factors. Patients were stratified into three risk groups with different survival outcomes as follows: low-risk group without any risk factors = low CDK7 protein level and high TIL score (*n* = 64); medium risk group with only one risk factor (*n* = 126); high-risk group with two risk factors = high CDK7 protein level and low TIL score (*n* = 32) (*P < 0.05). **k** Kaplan-Meier survival analysis of patients with different risk scores by CDK7 protein level, MYC protein level, and TILs score in cohort II. High CDK7 protein level, high MYC protein level and low TIL score were defined as three risk factors. Patients were stratified into three risk groups with different survival outcomes as follows: low-risk group without any risk factors (*n* = 41); medium risk group with only one risk factor (*n* = 97); high-risk group with at least two risk factors (*n* = 84) (**P < 0.01). **l** Schematic illustration showing the CDK7-p38α-MYC axis dependent regulation of PD-L1 and the function of this signaling in NSCLC. The solid arrows represent strong processes; the dashed arrows represent very weak processes after CDK7 inhibition

Therefore, we evaluated the functional importance of the regulation of PD-L1 expression by CDK7 in tumor progression and tested the combination of therapeutic blockade of CDK7 and PD-1 in vivo. In Lewis murine lung cancer model, THZ1 and antiPD-1 antibody combination therapy group showed a significant reduction in tumor burden than THZ1 or antiPD-1 alone treatment group (Fig. 6a–c) with no significant loss of body weight or other common toxic effects. Consistent with an intensified antitumor effect, immune profiling of Lewis lung cancer models demonstrated lower PD-L1 expression on tumor surfaces (Fig. 6d) and more tumor-infiltrating CD8^+^ T cells in mice receiving the combined THZ1 and antiPD-1 therapy (Fig. 6f). Specifically, the combination of THZ1 and antiPD-1 therapy could enhance the presence of CD45^+^ immune cells (Fig. 6e) and lead to a higher CD8^+^ T cell/CD45^+^ cell ratio (Fig. 6g). To be noted, interferon-γ (IFN-γ) was much higher in THZ1 and antiPD-1 combination treatment group than other groups in Lewis lung cancer tumor (Fig. 6h). Because checkpoint blockade therapies have obtained remarkable success in many cancers especially in melanoma, we verified that THZ1 sensitized tumor cells to antiPD-1 therapy by downregulating PD-L1 and recruiting tumor-infiltrating CD8^+^ T cells in B16 melanoma models (Figure S6A-G).

To confirm the role of PD-L1 in the combinatory therapeutic effects, we made a PD-L1-overexpressing Lewis cell line, and cell surface PD-L1 expression was confirmed by using flow cytometry (Figure S6J). We monitored the tumor growth of these tumors in vivo and found that the antitumor effect of the combined THZ1 and antiPD-1 therapy were impaired by PD-L1 overexpressing (Figure S6K-L). The results confirmed that the THZ1-induced PD-L1 downregulation played the main role in tumor regression in the combinatory treatment group. We then used the CD8^+^ T cell depletion antibody to assess the role of CD8^+^ T cell in THZ1-evoked antitumor immunity. As depicted in Fig. 6i, CD8^+^ T cell depletion impaired the antitumor effect of both THZ1 alone treatment and the combined THZ1 and antiPD-1 therapy. Moreover, THZ1 suppressed the p38α/MYC/PD-L1 axis (Figure S7A-B) and lactate production in vivo (Figure S7C). By CD8 staining via IHC, we showed that CD8 depletion antibody and PD-L1 over-expression impaired the THZ1 and antiPD-1 combinatory treatment-induced CD8^+^ T cell recruitment (Figure S7D-E). These data showed THZ1-suppressed PD-L1 expression in vivo and sensitized cancer cells to antiPD-1 therapy via recruiting infiltrating CD8^+^ T cells.

By defining high CDK7 expression and low tumor-infiltrating lymphocyte (TIL) score as two risk factors, patients in cohort II were stratified into three risk groups with different survival outcomes. The low-risk group with low CDK7 expression and high TIL score had a longer OS than the high-risk group with high CDK7 expression and low TIL score (Fig. 6j). When considering high MYC as a third risk factor, we showed that high-risk groups with at least two risk factors had worse survival outcomes than the low-risk group with no risk group in cohort II (Fig. 6k). These data showed that the CDK7-p38α-MYC signaling associated with TILs status could serve as prognostic predictors in NSCLC. In summary, our results showed that the CDK7-p38α-MYC axis-dependent regulation of PD-L1 plays a critical role in NSCLC and thus provided a promising strategy for applying THZ1 for boosting antitumor immunity of antiPD-1 therapy (Fig. 6l).

## Discussion

Recently, immunotherapy with checkpoint inhibition has become a major advancement in the treatment of NSCLC patients. The PD-1/PD-L1 pathway blockade therapies unleash the anti-tumor immune response. However, the response rates are around 20% in the majority of clinical trials [47]; there is a great need to find new combinatory treatments and increase efficacy. In this study, we have demonstrated that CDK7 inhibition in NSCLC cells downregulated PD-L1 expression via suppressing the p38α-MYC axis and boosts antitumor immunity of antiPD-1 therapy in vivo by recruiting infiltrating CD8+ T cells. The outstanding ability of CDK7 inhibitor THZ1 to convert PD-1 blockade-resistant tumors to PD-1 blockade-responsive tumors may provide a promising method to increase the efficacy of antiPD-1 antibodies.

The concept of transcriptional addiction refers to the behavior of specific subsets of cancers that show an absolute dependence on reprogrammed transcriptional programs during tumor progression [48]. In recent years, dysregulated TFIIH complex has been reported as a master regulatory machine in cancer progression of solid and hematologic malignancies [15, 49], and the main subunit CDK7 is recognized as a druggable target in cancer treatment. Here, we showed that selectively targeting CDK7 promoted apoptosis and suppressed NSCLC growth, which was validated in a lung adenocarcinoma PDX model. In line with recent work [15], we observed that CDK7 inhibitor THZ1 reduced phosphorylation of RNAPII CTD at S2, S5, and S7 sites in NSCLC cells, indicating effective inhibition of global transcription [7] which is confirmed by RNA-seq in THZ1-treated NSCLC cells. To investigate the mechanisms underlying the cytotoxic and transcriptional effects of THZ1 on NSCLC cells, we next performed gene expression profiling analysis. First, THZ1 treatment caused a broad reduction in transcription, and gene set enrichment analysis (GSEA) [30] showed that THZ1 influenced the function of many important oncogenes and transcriptional factors including MYC, STAT2/3/5, NRF2, EZH2, KLF4, NANOG, and GATA2 (Figure S8). MYC ranks as one of the top genes associated with THZ1-regulated genes, with a higher normalized enrichment score (ES). Our previous study and other researchers’ studies demonstrated that THZ1 suppressed cancer proliferation by disturbing MYC function [15, 34, 50]. We further analyzed the THZ1-regulated genes in the KEGG pathway database [29] and showed that THZ1-regulated pathways were enriched in three subsystems [25]: immune-related pathways, p38α-related pathways, and MYC-related pathways. Notably, both p38α [42] and MYC [24, 25] are involved in antitumor immunity by regulating PD-L1. Moreover, p38α could regulate MYC protein via AP1-REGγ-Wnt/beta-catenin signaling [40] and stabilize MYC mRNA via modulating TTP phosphorylation [41]. Thus, we hypothesized that the CDK7/p38α/MYC pathway may play an important role in NSCLC progression especially in immune evasion. Then our data showed that both CDK7 knockdown and THZ1 diminished p38α and MYC protein in NSCLC cells. Additionally, the upregulation and downregulation of p38α expressions caused the changes of MYC protein level in the same direction. Subsequently, p38α overexpression partially rescued THZ1-induced MYC ablation. Consistent with the published report [41], p38α knockdown and p38α inhibitor led to MYC mRNA destabilization, which explained why p38α inhibition decreased MYC protein level. Collectively, these findings suggested that CDK7 inhibition could suppress NSCLC growth via the p38α/MYC axis.

Because p38α has been reported as a promising therapeutic target in many cancers including NSCLC [31], we test if inhibition of CDK7 and p38α could enhance antitumor effects in synergy. Herein, we found that the combined treatment of THZ1 with p38α inhibitor LY2228820 caused synergistic effects in the induction of apoptosis and inhibition of tumor proliferation in vitro and in vivo. These results showed that the CDK7/p38α/MYC signaling offered us rational combination treatments for NSCLC. However, further investigation of the combination treatment in a broader panel of NSCLC cell lines and in vivo is warranted to determine the effectiveness of such combination treatment and potential selectivity-utility in an NSCLC context.

MYC has been reported to transcriptionally regulate PD-L1 expression to promote escape immunosurveillance [24]. Then, we validated the significance of CDK7-dependent regulation of MYC in remodeling tumor immune microenvironment. First, CDK7 inhibition decreased PD-L1 mRNA and protein levels in NSCLC. Subsequently, we verified MYC overexpression rescued PD-L1 decrease caused by CDK7 inhibition (Fig. 5). Moreover, MYC is a master regulator of tumor energy metabolism. In line with a recent study [51], we showed that THZ1 could suppress tumor glucose metabolism in NSCLC cells, which may depend on downregulating MYC-targeted genes including HK2, GLUT, and CDC25A. Tumor-derived lactate led to PD-L1 upregulation in lung cancer cells [44], and we found that THZ1 suppressed tumor-released lactate. Taken together, we conclude that CDK7 inhibition decreases PD-L1 expression by suppressing MYC function. Then, we showed that THZ1 sensitized tumors to antiPD-1 antibodies in vivo by decreasing PD-L1 expression and recruiting infiltrating CD8^+^ T cells which drive the antitumor immunity response [25]. A recent study showed that CDK7 inhibitor YKL-5-124 causes DNA damage in small cell lung cancer (SCLC) and sensitizes tumors to antiPD-1 therapy by provoking a robust immune surveillance program elicited by T cells [52]. In contrast to THZ1, YKL-5-124 treatment had no effect on CTD phosphorylation of RNA Pol II, indicating that YKL-5-124 does not inhibit global transcription. This may be the main reason for that THZ1 and YKL-5-124 boosted antitumor immunity by different mechanisms. Since p38α inhibition increases DNA damage and chromosome instability in breast cancer [31] and THZ1-regulated DNA homologous recombination repair associated genes such as BRCA1 and BRCA2 (Fig. 3b, i), the role of DNA damage in antitumor effects of THZ1 may need further exploration. These findings suggest a direct role of CDK7-dependent PD-L1 expression in mediating antiPD-1 therapy sensitivity. More importantly, there were no significant increased treatment-related toxicities in the combined therapies. However, the optimal drug doses and sequences in the combined treatments require more experiments to confirm in the future. We also need to apply new methods such as single-cell RNA-seq to analyze the direct effects of THZ1 on immune cells and stromal cells.

Previous studies showed that high CDK7 expression correlates with worse clinical outcome in multiple cancer types [53–56]. Our data showed that CDK7 is highly expressed in NSCLC tumor tissue, and high CDK7 expression is a poor prognostic predictor. Moreover, high CDK7 expression, high MYC expression, and low tumor-infiltrating lymphocytes (TILs) could serve as three risk factors to differentiate NSCLC patients with different survival outcomes. In summary, our work provides preclinical evidence for the combined THZ1 and antiPD-1 therapy in future NSCLC clinical trials.

## Supplementary information

**Additional file 1:.** Supplementary file

## Data Availability

All data generated or analyzed during this study are included in this published article and its supplementary information files or from the corresponding author upon reasonable request.

## Abbreviations

CDK7: Cyclin-dependent kinase 7
MAPK14: Mitogen-activated protein kinase 14
MYC: v-myc avian myelocytomatosis viral oncogene homolog
NSCLC: Non-small cell lung cancer
Pol II: RNA polymerase II
CTD: C-terminal domain
HK2: Hexokinase 2
ICBs: Immune checkpoint blockades
TMAs: Tissue microarrays
TCGA: The Cancer Genome Atlas
CCLE: The Cancer Cell Line Encyclopedia
TCPA: The Cancer Proteome Atlas
IHC: Immunohistochemistry
TILs: Tumor-infiltrating lymphocytes
IF: Immunofluorescence
GO: Gene Ontology
PDX: Patient-derived xenograft
LLC: Lewis lung carcinoma
s.c.: Subcutaneously
i.p.: Intraperitoneally
HBSS: Hank’s Balanced Salt Solution
RT: Room temperature
MFI: Mean fluorescence intensity
SD: Standard deviation
ANOVA: One-way analysis of variance
CI: Combination index
DFS: Disease-free survival
OS: Overall survival
SCLC: Small cell lung cancer
TTP: Tristetraprolin
PBMC: Peripheral blood mononuclear cell
IFN-γ: Interferon-γ.
