# Supplementary material for: CDK7 inhibitor THZ1 enhances antiPD-1 therapy efficacy via the p38α/MYC/PD-L1 signaling in non-small cell lung cancer
Source: J Hematol Oncol. 2020 Jul 20;13:99. doi: 10.1186/s13045-020-00926-x (PMC7370470; doi:10.1186/s13045-020-00926-x)
Supplement: Supplementary file 1 — Additional file 1:. Supplementary file [file 13045_2020_926_MOESM1_ESM.docx]

**CDK7 inhibitor THZ1 enhances antiPD-1 therapy efficacy via the p38α/MYC/PD-L1 signaling in non-small cell lung cancer**

Jian Wang^1,#^, Ruiguang Zhang^1,#^, Zhenyu Lin^1^, Sheng Zhang^1^, Yaobing Chen^2^, Jing Tang^1^，Jiaxin Hong^1^, Xiaoshu Zhou^1^, Yan Zong^1^, Yingzhuo Xu^1^, Rui Meng^1^ , Shuangbing Xu^1^, Li Liu^1^, Tao Zhang^1^, Kunyu Yang^1^, Xiaorong Dong^1，*^, Gang Wu^1，*^

^1^Cancer Center, Union Hospital, Tongji Medical College, Huazhong University of Science and Technology, Wuhan 430022, China.

^2^Institute of Pathology, Tongji Hospital, Tongji Medical College, Huazhong University of Science and Technology, Wuhan 430030, China

**# These authors contribute equally to this work.**

*** Corresponding author:** Cancer Center, Union Hospital, Tongji Medical College, Huazhong University of Science and Technology, Wuhan 430022, China.

X. Dong (xiaorongdong@hust.edu.cn) and G. Wu (xhzlwg@163.com)

**Supplementary Figures**


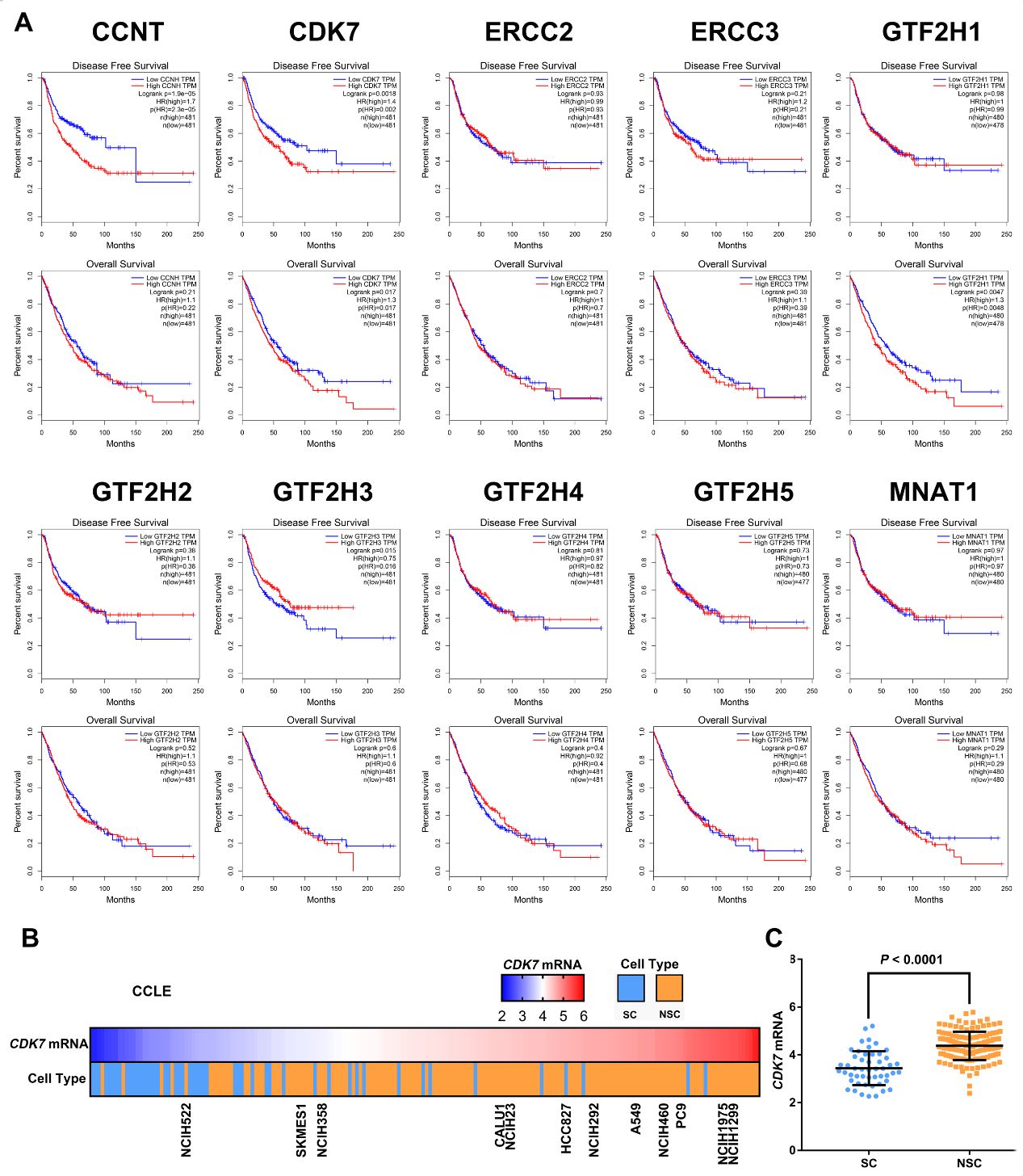


### Figure S1. Analysis of the relationship between the 10-subunit TFIIH complex mRNA levels and survival outcomes in NSCLC.

(A) Kaplan-Meier survival curves (K-M curves) showing the relationship between 10 subunits of TFIIH complex mRNA levels and survival outcomes in the TCGA NSCLC dataset by using GEPIA (<http://gepia.cancer-pku.cn/>). A median gene mRNA expression cut-off point was used for stratification. (B) Heatmap showing *CDK7* mRNA expression in SCLC and NSCLC cell lines. Data were extracted from CCLE (https://portals.broadinstitute.org/ccle). (C) Data mining of CCLE showing differential *CDK7* mRNA levels in SCLC and NSCLC cell lines. (*P* < 0.0001)


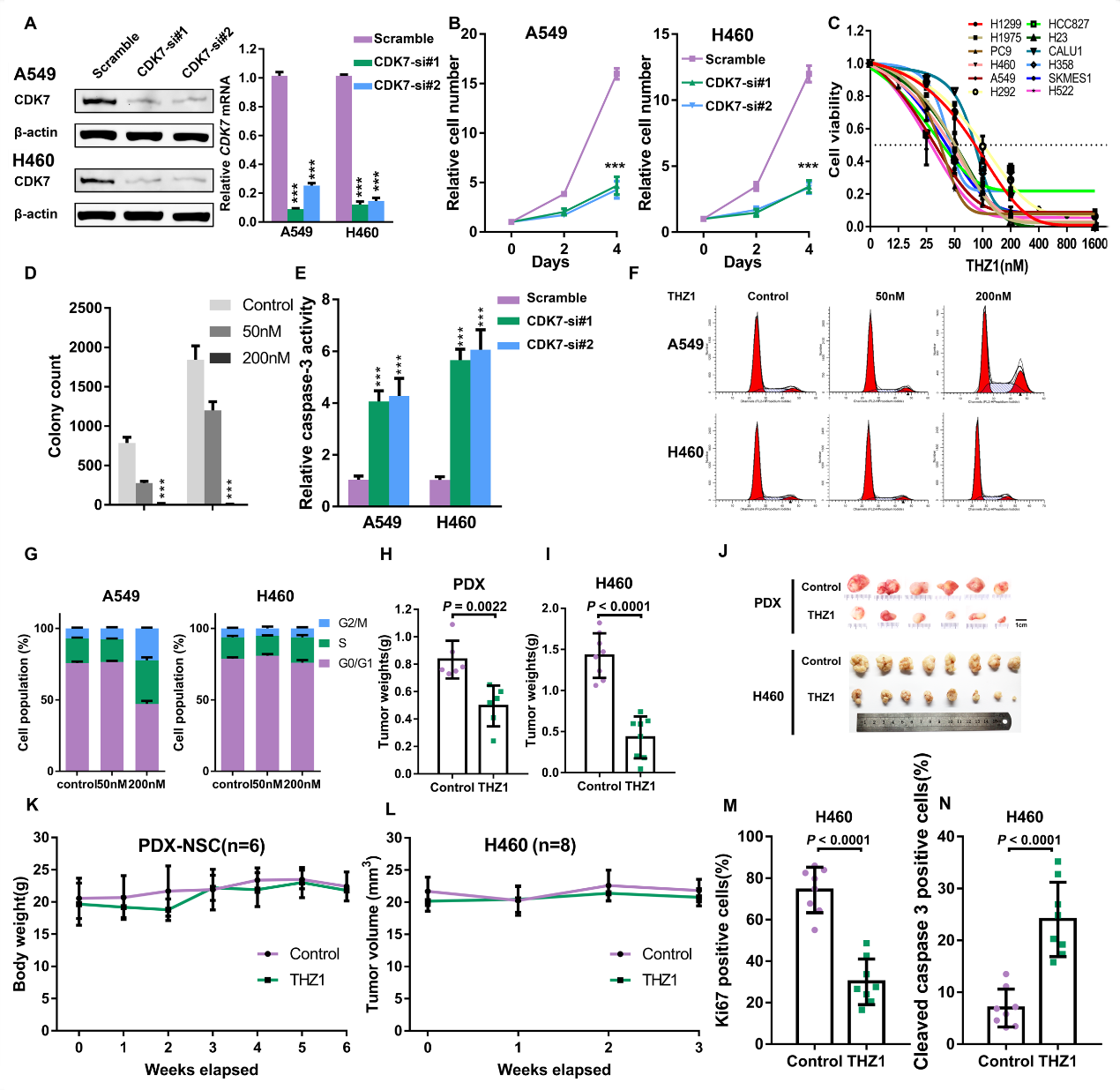


### Figure S2. THZ1 promotes apoptosis and suppresses NSCLC growth in vitro and in vivo.

(A) Validation of CDK7 knockdown by Western blot and PCR (n = 3) (****P* < 0.001 as compared to scramble group). (B) CDK7 silencing suppressed cell growth. Cells were transfected with CDK7 siRNAs for 48 h and then seeded in 6-well plates. Cell numbers were counted at indicated time (n = 3) (****P* < 0.001 as compared to scramble group). (C) Representative NSCLC cell lines were exposed to increasing doses of THZ1 at 72 h (n = 3). (D) Quantification of colony formation in A549 and H460 cells treated with vehicle or THZ1 (n = 3) (****P* < 0.001 as compared to vehicle group). (E) Caspase-3 activity was measured after NSCLC cells were transfected with scramble or CDK7 siRNAs. Results are presented as fold-increase to scramble group (n = 3) (****P* < 0.001 as compared to scramble group). (F) Representative images of cell cycle analysis by PI staining in NSCLC cells treated with vehicle or THZ1(48 h). (G) Quantification of percentages of A549 and H460 cells in different cell cycle phases following treatment with various doses of THZ1 for 48 hours (n = 3). (H) Weights of tumors from mice in the PDX model at the endpoint (n = 6) (*P* = 0.0022). (I) Weights of tumors from mice in H460 xenograft models at the endpoint (n = 8) (*P* < 0.0001). (J) Photographs of tumors from both vehicle and THZ1 (twice daily, 10 mg/kg, 3 weeks) treatment groups in the PDX models (up; pictured separately) and H460 xenograft models (down). (K) Bodyweight changes of mice in PDX models during vehicle or THZ1 treatment (Not significant). (L) Body weight changes of mice in H460 xenograft models during vehicle or THZ1 treatment (Not significant). (M) Quantification of ki67 positive cells by IHC staining in tumor tissue sections from H460 xenograft at the endpoint (n = 8) (*P* < 0.0001). (N) Quantification of cleaved Caspase-3 positive cells by IHC staining in tumor tissue sections from H460 xenograft at the endpoint (n = 8) (*P* < 0.0001).


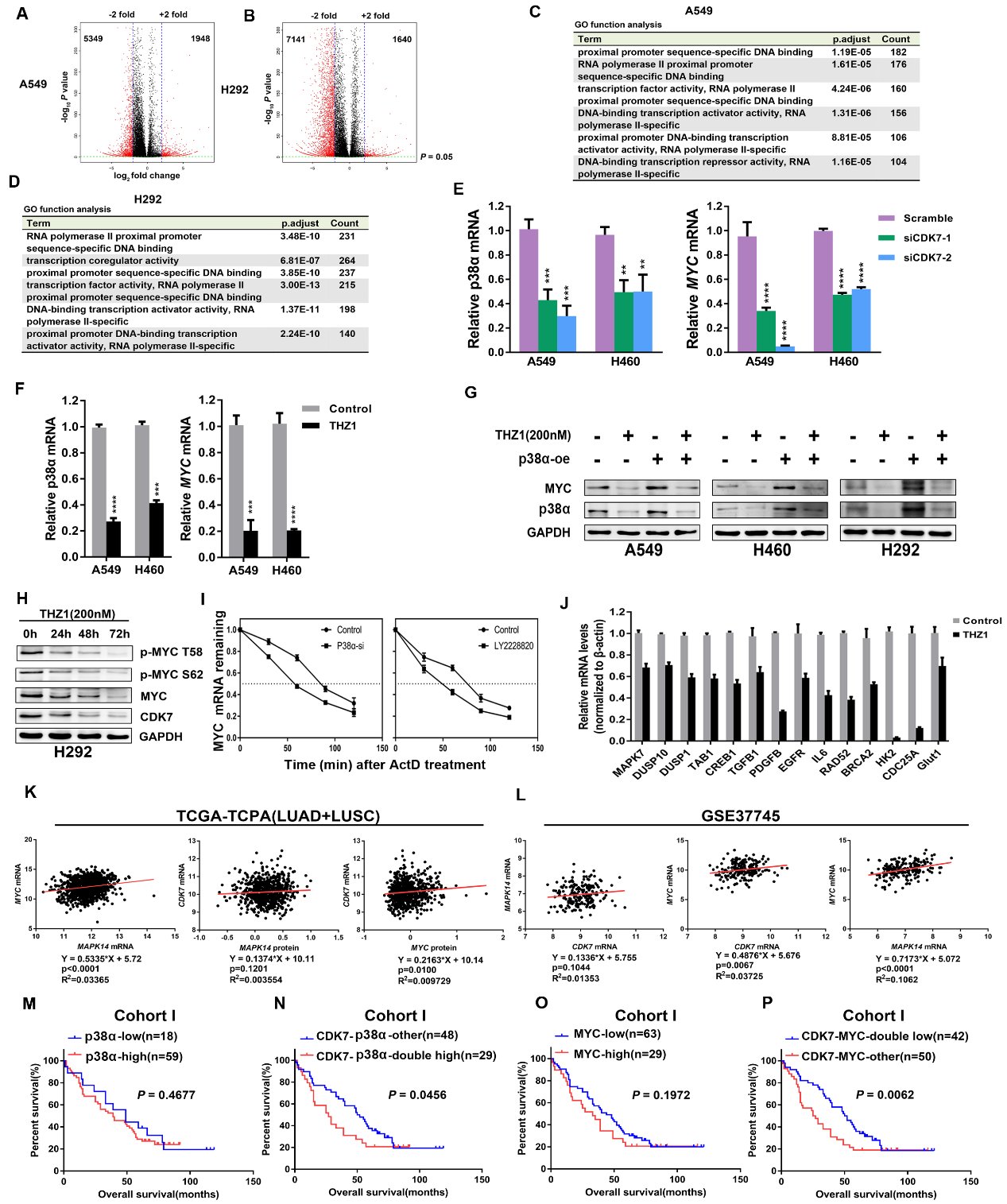


**Figure S3 THZ1 inhibits p38α/MYC pathway in NSCLC.**

(A) Volcano plots showing expression changes (log2 fold) of all active transcripts in A549 cells upon either vehicle or THZ1 treatment (200 nM, 24 h). (B) Volcano plots showing expression changes (log2 fold) of all active transcripts in H292 cells upon either vehicle or THZ1 treatment (200 nM, 24 h). (C) GO analysis of differential expression genes for THZ1 (200nM, 24 h) treated cells over vehicle in A549 cells. Lists were ranked by counts in each GO functional category. (D) GO function analysis of differential expression genes for THZ1 (200nM, 24 h) treated cells over vehicle in H292 cells. Lists were ranked by counts in each GO functional category. (E) Quantitation of relative p38α and MYC mRNA expression in NSCLC cell lines transfected with either scramble or CDK7 siRNAs. Results are normalized to β-actin (n = 3) (***P* < 0.01; ****P* < 0.001; *****P* < 0.0001 as compared to scramble group). (F) Quantitation of relative p38α and MYC mRNA expression in NSCLC cell lines treated with vehicle or THZ1(200nM, 24h). Results are normalized to β-actin (n = 3) (****P* < 0.001; *****P* < 0.0001 as compared to control group). (G) Immunoblot of MYC proteins after NSCLC cells treated with THZ1 (200nM) or transfected with p38α (encoded by MAPK14) vector as well as the combination. GAPDH was used as a loading control. (H) Immunoblot of MYC proteins in H292 cells after THZ1 (200nM) treatment at the indicated time. (I) MYC mRNA levels in A549 cells with p38α inhibited by siRNA or LY2228820 after Actinomycin D (ActD) treatment over the specified time. (J) Quantitation of relative mRNA changes in NSCLC cell lines treated with vehicle or THZ1(200nM, 24h). Results are normalized to β-actin (n = 3). (K) Correlation among CDK7, p38α and MYC expression in TCGA-TCPA NSCLC data. (L) Correlation among CDK7, p38α and MYC mRNA levels in GSE37745 data. (M) Kaplan-Meier survival analysis of patients with high or low p38α protein level in Cohort I (n = 77) (*P* = 0.4677). (N) Kaplan-Meier survival analysis of patients stratified by CDK7 and p38α protein levels in Cohort I. High CDK7 and p38α protein levels were defined as two risk factors. Patients were stratified into two risk groups with different survival outcomes as follows: patients with CDK7 and p38α double high protein level (n = 29) had a shorter OS than the others (n = 48) in Cohort I (P = 0.0456). (O) Kaplan-Meier survival analysis of patients with high or low MYC protein level in Cohort I (n = 92) (*P* = 0.1972). (P) Kaplan-Meier survival analysis of patients stratified by CDK7 and MYC protein levels in Cohort I. High CDK7 and MYC protein levels were defined as two risk factors. Patients were stratified into two risk groups with different survival outcomes as follows: patients with CDK7 and MYC double low protein level (n = 42) had a longer OS than the others (n = 50) in Cohort I (*P* = 0.0062).

**
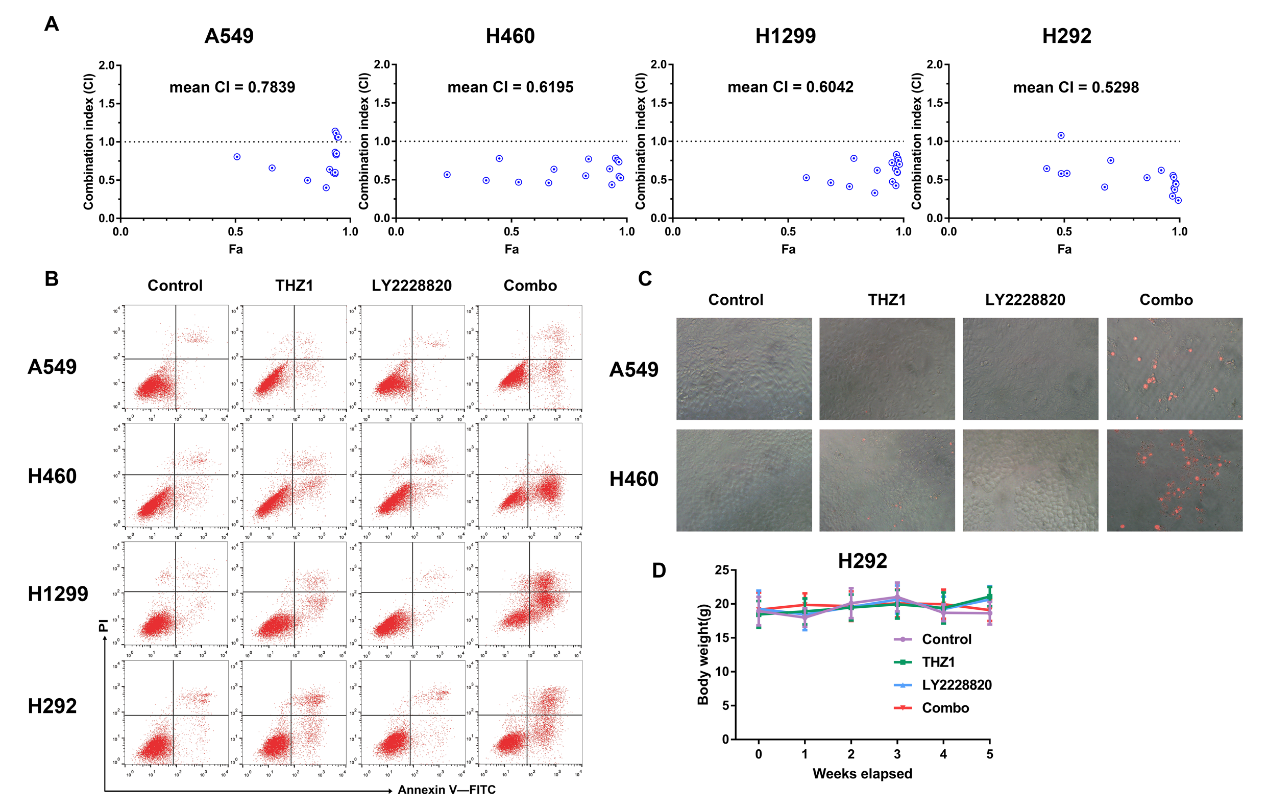
**

**Figure S4. THZ1 increased NSCLC sensitivity to LY2228820.**

(A) Combination index (CI) calculation for THZ1 and LY2228820 in A549, H460, H1299 and H292 cells by using the CompuSyn method. All NSCLC cells were measured after 72 h of drug treatments by CCK8 cell viability assay (n = 3). CI value of less than 0.9 was significant. (B) Representative images of Annexin V/propidium iodide staining after the combination of THZ1 (50 nM) and LY2228820 (5μM) for 48 h (n = 3). (C) Representative images showing TUNEL positive cells (with nucleus stained in red) after the combination of THZ1 (50 nM) and LY2228820 (5 μM) for 48 h (n = 3, original magnification × 400). (D) Body weight changes of mice in H292 xenograft models during the combination treatment of THZ1 and LY2228820 (n = 6) (Not significant).


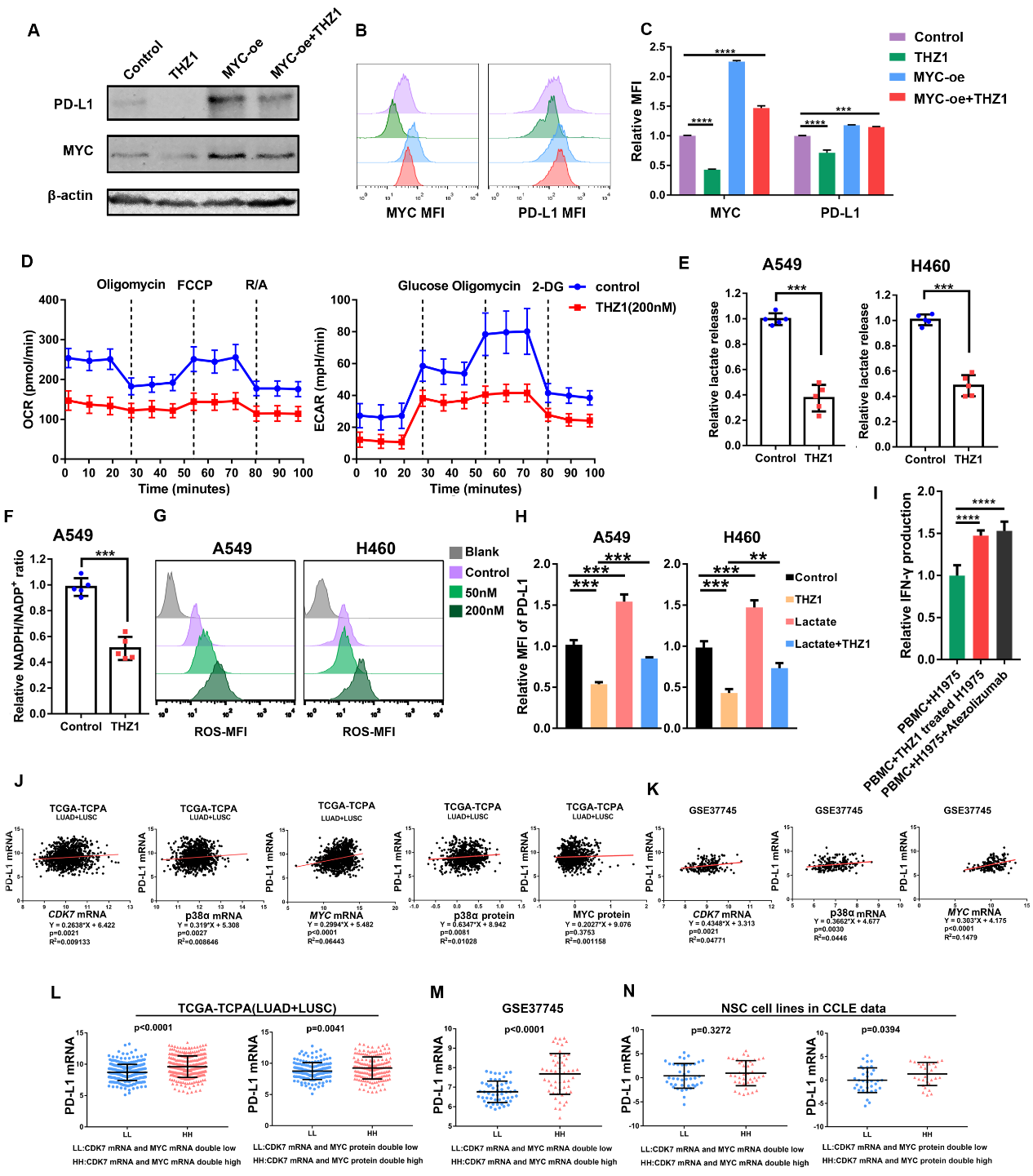


**Figure S5. CDK7 suppresses PD-L1 expression in NSCLC.**

(A) Immunoblot of MYC and PD-L1 proteins after A549 cells transfected with MYC vector or treated with THZ1. β-actin was used as a loading control. (B) Representative images showing PD-L1 detection on cell surfaces and MYC detection in cells at the same time in A549 cells transfected with MYC vector or treated with THZ1. (C) Quantitation of PD-L1 detection on cell surfaces and MYC detection in cells at the same time in A549 cells transfected with MYC vector or treated with THZ1. (n = 3) (****P* < 0.001; *****P* < 0.0001). (D) Extracellular acidification rate (ECAR, left) and Oxygen consumption rate (OCR, right) was measured in A549 after THZ1 (200 nM) treatment (n = 5). (E) Quantitation of lactate release alternation after THZ1 treatment in A549 cells at 24 h (n = 5) (****P* < 0.001). (F) Quantitation of NAPDH/NAPD+ ratio changes after THZ1 treatment in A549 cells at 24 h (n = 5) (****P* < 0.001). (G) Representative FACS images of ROS detection by H2DCFDA staining in A549 and H460 cells treated with either vehicle or THZ1 at 24 h. (H) Quantitation of PD-L1 detection on surfaces of NSCLC cell lines treated with THZ1 (200 nM) or stimulated by lactate (15 μM) (n = 3) (***P* < 0.01; ****P* < 0.001). (I) IFN-γ production in the PBMC-NSCLC coculture system. (n=5) (*****P* < 0.0001). (J) Correlation between PD-L1 mRNA level and the CDK7-p38α-MYC axis in TCGA-TCPA NSCLC data. (K) Correlation between the PD-L1 mRNA level and the CDK7-p38α-MYC axis in GSE37745 data. (L) Patients with CDK7 and MYC double high mRNA levels had a higher PD-L1 mRNA level than patients with CDK7 and MYC double low based on TCGA-TCPA NSCLC data. (M) Patients with CDK7 and MYC double high mRNA levels had a higher PD-L1 mRNA level than patients with CDK7 and MYC double low based on GSE37745 data. (N) NSCLC cell lines with CDK7 and MYC double high mRNA level had a higher PD-L1 mRNA level than cells with CDK7 and MYC double low based on CCLE data.


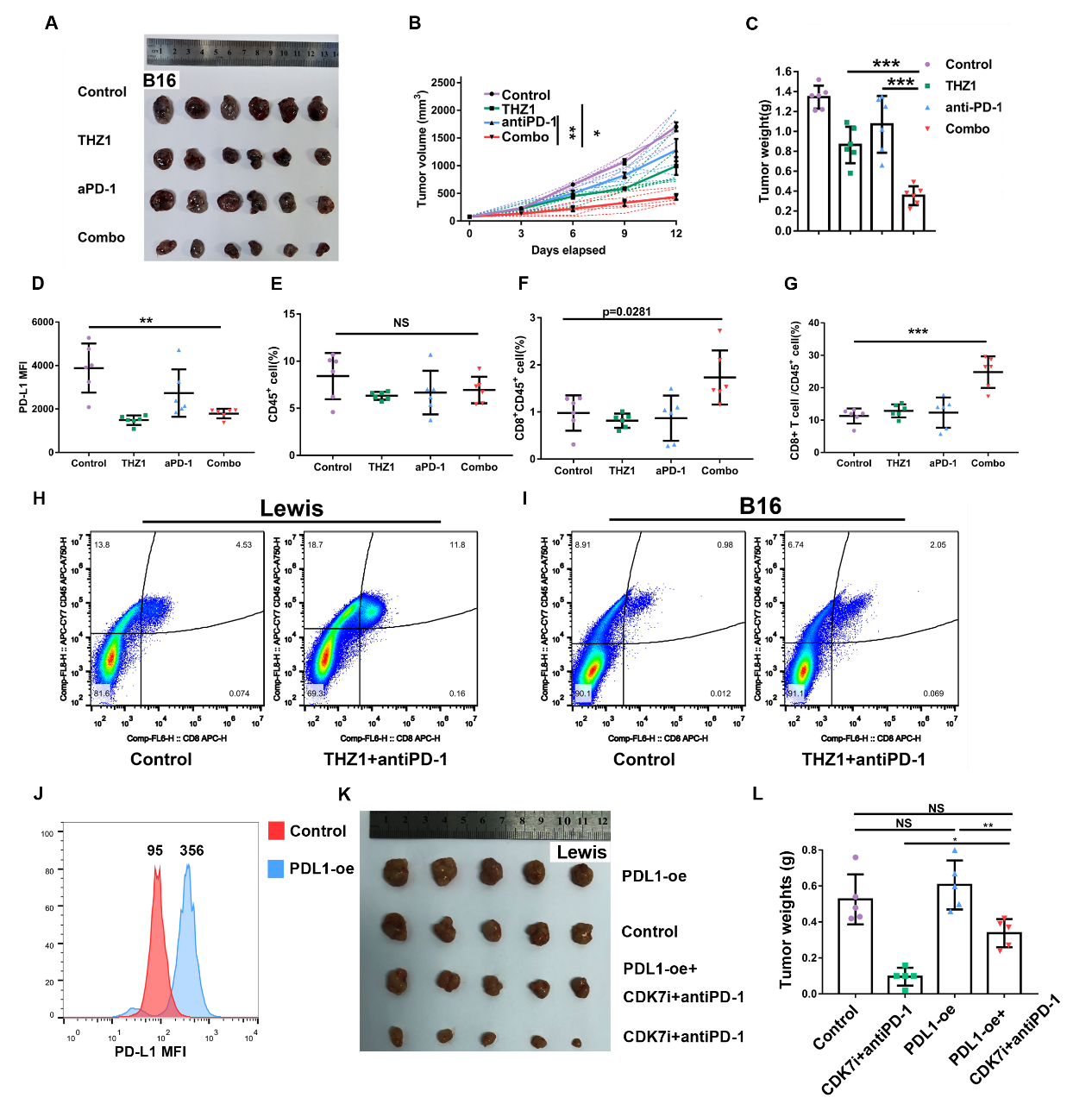


**Figure S6. CDK7 inhibition overcomes tumor resistance to PD-1 blockade.**

(A) Photographs of tumors from the B16 murine melanoma model treated with the combination of THZ1 and antiPD-1 antibody (n = 6). (B) Tumor growth curves of mice from the B16 model. The dashed line represents a single mouse data in each group and the solid line represents the mean value in different groups. Error bars represent ± SEM (**P* < 0.05; ***P* < 0.01). (C) Weights of tumors from mice in B16 model at the endpoint (**P* < 0.05; ****P* < 0.001). (D) Quantitation of PD-L1 expression on tumor surface from B16 model (****P* < 0.001). (E) Quantification of percentages of CD45^+^ cells in tumors from the B16 model treated with the combination of THZ1 and antiPD-1 antibody (****P* < 0.001). (F) Quantification of percentage of CD45^+^CD8^+^ cells in tumor from B16 model treated with the combination of THZ1 and antiPD-1 antibody (n = 6) (***P* < 0.01; ****P* < 0.001). (G) CD8^+^ T cell/CD45^+^ cell ratio in tumor from B16 model (****P* < 0.001). (H) Representative images of infiltrating lymphocyte analysis gated by CD45 and CD8 levels in the Lewis lung cancer model. (I) Representative images of infiltrating lymphocyte analysis gated by CD45 and CD8 levels in the B16 melanoma model. (J) Detection of PD-L1 levels on cell surfaces from Lewis lung cancer transfected with PD-L1 vector. (K) Photographs of tumors from the Lewis lung cancer model with PD-L1 overexpressed treated with the combination of THZ1 and antiPD-1 antibody (n = 5). (L) Weights of tumors from Lewis lung cancer model with PD-L1 overexpressed treated with the combination of THZ1 and antiPD-1 antibody at the endpoint (n = 5) (**P* < 0.05; ***P* < 0.01).


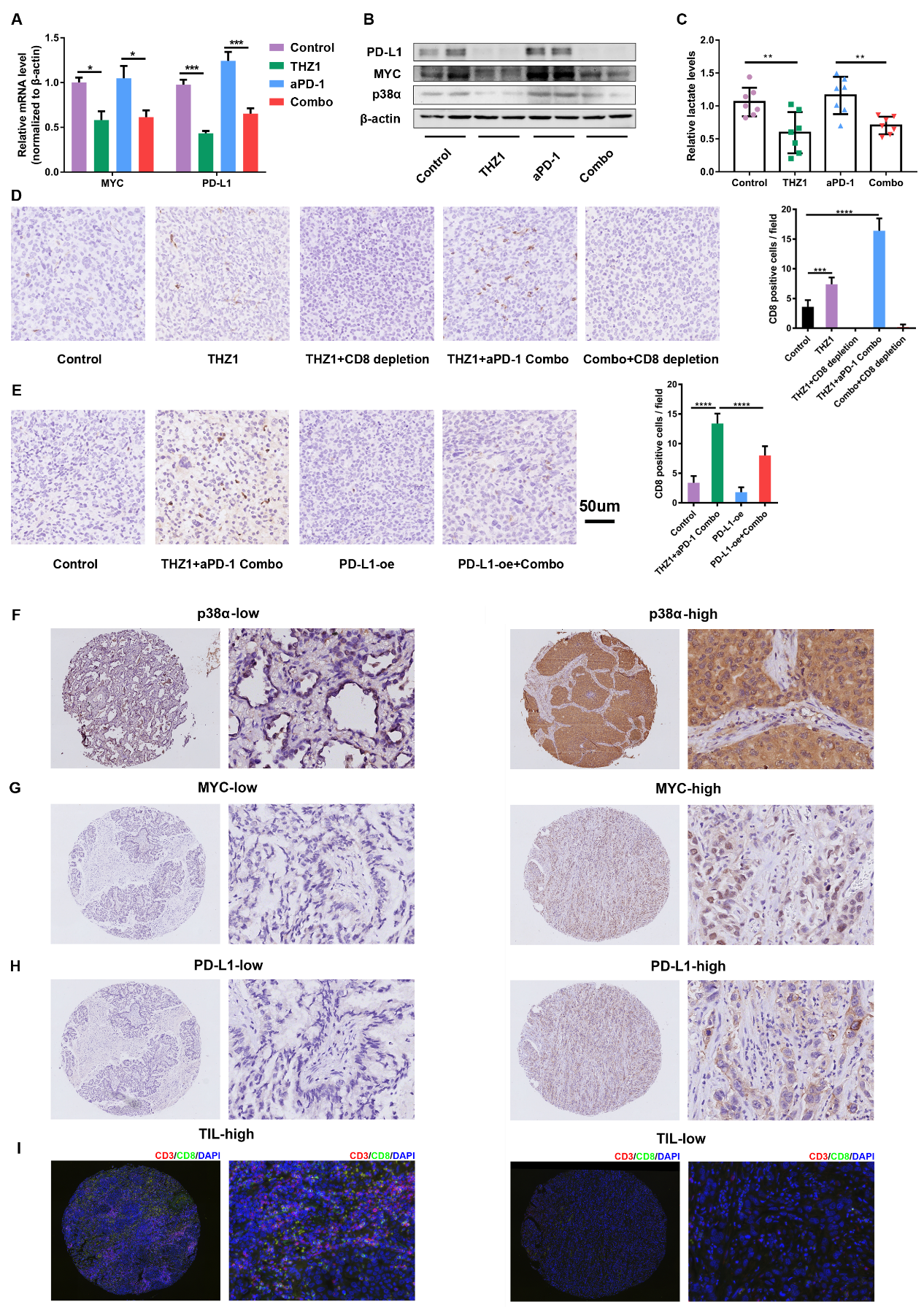


**Figure S7. Changes of** **the p38α/MYC/PD-L1 axis and T cell infiltration in Lewis models.**

(A) Changes of MYC and PD-L1 mRNA levels in Lewis tumor after the combination of THZ1 and antiPD-1 treatment (**P* < 0.05; ****P* < 0.001). (B) Immunoblot of p38α, MYC and PD-L1 proteins in Lewis tumor after the combination of THZ1 and antiPD-1 treatment. β-actin was used as a loading control. (C) Quantitation of lactate concentration in Lewis tumor after the combination of THZ1 and antiPD-1 treatment (***P* < 0.01). (D) Representative images (left; scale bar, 50 μm) of Lewis tumor tissue stained with CD8 antibody by immunohistochemistry (IHC) in the CD8^+^ T cell depletion experiment and the quantification of CD8 positive cells (right; ****P* < 0.001, *****P* < 0.0001). (E) Representative images (left; scale bar, 50 μm) of Lewis tumor tissue stained with CD8 antibody by IHC in the PD-L1 over-expression rescue experiment and the quantification of CD8 positive cells (right; *****P* < 0.0001). (F-I) Representative scanned images of tissue cores in Cohort I and II with low or high p38α (F), MYC (G), and PD-L1 (H) by IHC as well as tumor-infiltrating lymphocytes (I) by immunofluorescence. Left, Original magnification, ×6; scale bar, 500 μm. Right, Original magnification, ×400; scale bar, 50 μm.

**
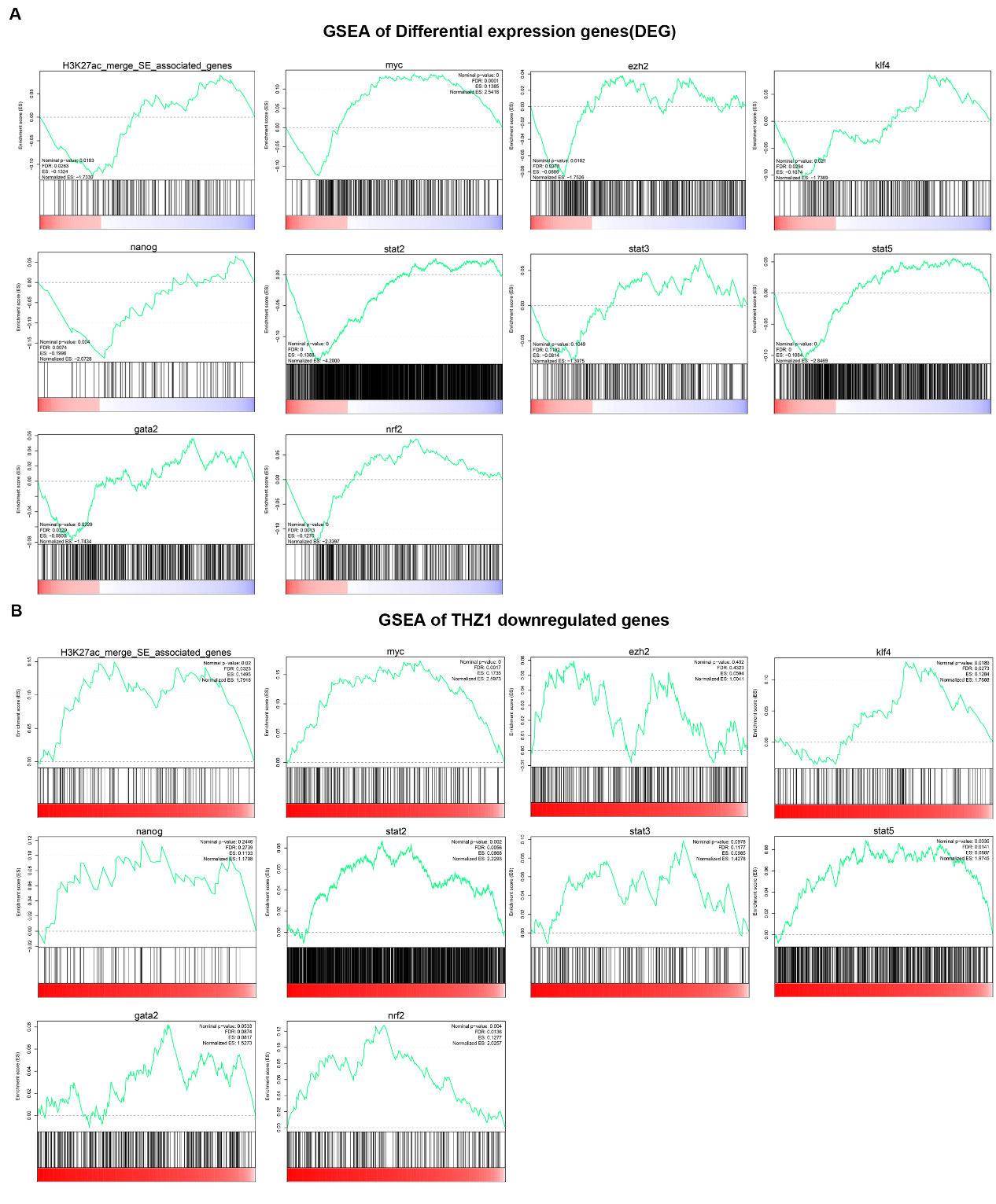
**

**Figure S8.** **Analysis of THZ1 regulated genes by Gene set enrichment analysis (GSEA).**

(A) Analysis THZ1 regulated genes (DEGs) by GSEA. (B) Analysis of THZ1 downregulated genes by GSEA.

**Supplementary methods**

**Cell lines and cell culture**

Cell lines were obtained originally from ATCC or collaborating labs primarily in 2014 and authenticated by suppliers. The most recent re-authentication of A549, H460, H1299, HCC827, H23, H1975 cells were was completed from January 2016 to May 2018. All cell lines were cultured in recommended medium supplemented with 10% heat-inactivated fetal bovine serum (FBS) and 1% penicillin/streptomycin, at 37 ℃ in a humidified incubator containing 5% CO_2_. Cells were mycoplasma tested monthly and passaged for less than 3 months.

## Reagents

THZ1 and Atezolizumab were purchased from Selleck. LY2228820 was purchased from MedChemExpress. Drugs were resuspended according to instructions and stored in aliquots at −80°C. The drugs were added to the culture medium and homogenized at different final concentrations before being added to the cell culture. All controls were normalized by adding the same amount of vehicle solution. Rat antiPD-1 IgG2a (RMP1-14) was purchased from BioXCell (#BE0146) and rat IgG2a isotype control antibody was purchased from BioXCell (#BE0089).

## Gene knockdown and overexpression

All siRNAs were transfected into cells at 100 nM using Lipofectamine RNAimax (Invitrogen) following the manufacturer’s protocol. Cells were harvested 48 h after transfection. The knockdown efficiency of the siRNAs was assessed by RT-PCR and Western blot. The sequences of siRNAs were as follows: CDK7 siRNA#1: 5′- GGAUGGCUCUGGACGUGAATT-3′；CDK7 siRNA#2: 5′-CGGAUAUCGGGAAGUUCAATT-3′；p38α (encoded by *MAPK14*) siRNA：5′-CAGUGAUACGUACAGCCAATT-3′；p38α and MYC plasmids were purchased from Beijing Syngentech and transferred into cells using Lipofectamine 2000 according to the manufacturer’s instructions.

## Cell viability assay

Cells were cultured in 96-well plates at 3-5 × 10^3^ cells per well and allowed to adhere overnight. The working solutions of agents were added to the culture medium for the indicated time and then cell viability was detected by CCK-8 kit (Sigma-Aldrich, USA) according to the manufacturer’s instructions. All experiments were done with 4–6 wells per experiment and repeated at least three times. Results were normalized to a vehicle-only control. IC50 was determined by four-parameter dose-response analysis. In combination analysis, synergy was measured using the CompuSyn software and showing by GraphPad Prism.

## Cell growth and Colony formation assay

For cell growth assay, cells transfected with the indicated siRNAs were seeded into six-well plates at 1-5 × 10^4^ cells/well. Cell numbers were counted at the indicated time by digesting into suspension with trypsin. For colony formation assay, cells were seeded at 1 x 10^4^ cells/well in 10-mm plates or 3-5 x 10^2^ cells/well in 6-well plates overnight and then treated with different regimens until colonies were visible. Colonies were stained with crystal violet solution and counted manually.

**Caspase-3 activity assay**

Caspase-3 activities were determined by Caspase-3 Activity Assay Kit (Beyotime Biotechnology, China) according to the manufacturer’s instructions. After treatment, cell lysates were prepared and Caspase-3 activities were evaluated by the ability of Caspase-3 to change an acetyl-Asp-Glu-Val-Asp p-nitroanilide (Ac-DEVD-pNA) into a yellow formazan product [p-nitroaniline (pNA)]. 40 μl reaction buffer, 50μl cell lysate and 10μl Ac-DEVD-pNA(2mM) were added by turns and then incubated at 37 ℃ for 1 h. Samples were measured at an absorbance of 405 nm. Protein content was measured according to Bradford assay as the internal control. All the experiments were carried out in triplicates.

**TUNEL assays**

Drug-treated cells were detected by TUNEL assays using the one-step TUNEL detection kit (Beyotime Biotechnology, China) according to the manufacturer's instructions. TUNEL^+^ cells (cells with a red nucleus) were evaluated in a randomly selected field of view at × 40 magnification. The results were expressed as the ratio of TUNEL^+^ cells to the total number of cells and normalized to the control group. All samples were assayed in triplicate.

**Quantitative interferon (IFN)-γ**

The collected tumors in Lewis models were harvested and part of the tumor tissue was weighed and homogenized for IFN-γ detection by using Quantikine ELISA (R&D Systems) according to instructions. The IFN-γ levels were normalized to the mass of tissue.

## Real-time PCR.

RT-PCR was done as previously described [[1](#_ENREF_1)]. Total RNA was isolated with PrimeScript™ RT Master Mix (Takara). The reverse transcription reactions were conducted with SYBR® Premix Ex Taq™ II (Takara). Real-time PCR was performed with a StepOne Plus Real-Time PCR System (Applied Biosystems). The human primer sequences were as follows: CDK7 forward 5′- GGGCAAAGCGTTATGAGAAGC-3′ and reverse 5′- AATGGCGACAATTTGGTTGGTG-3′, p38α (*MAPK14*) forward 5′- GAGGTGCCCGAGCGTTAC-3′ and reverse 5′- GGACTGAAATGGTCTGGAGAGC-3′, HK2 forward 5′- CGTGGAACTGGTGGAAGGAG-3′ and reverse 5′- CTGTGCGGAAGTCATCTAGGC-3′, CDC25A forward 5′- GTTTGACTCCCCTTCCCTGTG-3′ and reverse 5′- CCAGACATGCTCTTCCTCCTC-3′, GLUT1 forward 5′- TGTGCTCCTGGTTCTGTTCTTC-3′ and reverse 5′- GCTCCTCGGGTGTCTTGTC-3′, PD-L1 forward 5′- CATGTCAGGCTGAGGGCTAC-3′ and reverse 5′- TGGAATTGGTGGTGGTGGTC-3′, MYC forward 5′-CTGCTTAGACGCTGGATTT-3′ and reverse 5′-TCGTAGTCGAGGTCATAGTTC-3′, GAPDH forward 5′-ACCACAGTCCATGCCATCAC-3′ and reverse 5′-TCCACCACCCTGTTGCTGTA-3′, β-actin forward 5′-GTCCACCGCAAATGCTTCTA-3′, reverse 5′-TGCTGTCACCTTCACCGTTC-3′, MAPK7 forward 5′- GTCTTCCATGTCAGAGTCACCTG-3′ and reverse 5′- CCAGGTCAAAGCCAACACCGTA-3′, DUSP10 forward 5′- CAGCCACTTCACATAGTCCTCG-3′ and reverse 5′- TGGAGGGAGTTGTCACAGAGGT-3′, DUSP1 forward 5′- CAACCACAAGGCAGACATCAGC-3′ and reverse 5′- GTAAGCAAGGCAGATGGTGGCT-3′, TAB1 forward 5′- GCTTCTTGGTGCTGATGTCGGA-3′ and reverse 5′- GAGGTCTGCTTGGCAAACTCAG-3′, TGFB1 forward 5′- TACCTGAACCCGTGTTGCTCTC-3′ and reverse 5′- GTTGCTGAGGTATCGCCAGGAA-3′, PDGFB forward 5′- GAGATGCTGAGTGACCACTCGA-3′ and reverse 5′- GTCATGTTCAGGTCCAACTCGG-3′, EGFR forward 5′- AACACCCTGGTCTGGAAGTACG-3′ and reverse 5′- TCGTTGGACAGCCTTCAAGACC-3′, IL6 forward 5′- AGACAGCCACTCACCTCTTCAG-3′ and reverse 5′- TTCTGCCAGTGCCTCTTTGCTG-3′ and RAD52 forward 5′- GCCCAGAATACATAAGTAGCCGC-3′ and reverse 5′- CCACATTCTGCTGCGTGATGGA-3′.

The mouse primer sequences were as follows: MYC forward 5′- CCTAGTGCTGCATGAGGAGA-3′ and reverse 5′- TCTTCCTCATCTTCTTGCTCTTC-3′, PD-L1 forward 5′- GCTCCAAAGGACTTGTACGTG-3′ and reverse 5′- TGATCTGAAGGGCAGCATTTC-3′, β-actin forward 5′- CGGTTCCGATGCCCTGAGGCTCTT -3′ and reverse 5′- CGTCACACTTCATGATGGAATTGA-3′. Samples were normalized to β-actin or GAPDH mRNA to determine relative expression.

## Western blot

Western blot was done as previously described[[1](#_ENREF_1)]. RIPA lysis buffer was used for protein extraction according to manufacturer instructions and protein concentration was determined by Bradford method. Equal proteins were submitted to SDS-PAGE electrophoresis and transferred onto PVDF membranes. After blocked in 5% skim milk for 1 h, membranes were probed with primary antibodies overnight at 4 ℃, followed by incubation with horseradish peroxidase–linked secondary antibodies for 1 hour at room temperature(RT), and visualized with an enhanced chemiluminescence reagent. The following primary antibodies were used: CDK7 (WB, 1:1000, CST #2916), RNAPII C-terminal domain (CTD) (WB, 1:1000, CST #14958), p-CTD (Ser2) (WB, 1:1000, CST #13499), p-CTD (Ser5) (WB, 1:1000, CST #13523), p-CTD (Ser7) (WB, 1:1000, CST #13780), cleaved Caspase-3 (WB, 1:1000, CST #9661), cleaved PARP (WB, 1:1000, CST #5625), p-MYC T58 (WB, 1:500, ABclonal #AP0080), p-MYC S62 (WB, 1:1000, ABclonal #AP0082), p-MYC (T58 + S62) (WB, 1:3000 dilution, Abcam #ab10568), MYC (WB, 1:1000, CST # 5605 and ABclonal #A11029), p38α (WB, 1:500, ABclonal #A14401), HK2 (WB, 1:500, ABclonal #A0994), PD-L1(WB, 1:1000, CST #13684 and ABclonal #A1645), GAPDH (WB, 1:3000 dilution, Abcam #ab8245) and β-actin (WB, 1:3000, Abcam #ab8227).

## Flow cytometry analysis of apoptosis and mitochondrial membrane potential

Apoptosis was detected using an Annexin V/propidium iodide staining detection kit (Beyotime Biotechnology, China). Cells were harvested and single-cell suspensions were made using 0.25 % trypsin without EDTA. Cell suspensions were washed with PBS twice and resuspended in 250 μL binding buffer at a density of 5 x 10^6^ cells/mL. The cell suspension was analyzed by flow cytometry after they were stained with 5 μL Annexin V-FITC and 5μL PI solution. Data were analyzed and shown by FlowJo software. JC-1 staining (Beyotime Biotechnology, China) was performed according to the manufacturer’s instructions to measure mitochondrial membrane potential as described previously[[1](#_ENREF_1)].

**MYC transcriptional activity**

Luciferase reporter assay system was assessed MYC transcriptional activity using pMyc-TA-luc (Beyotime, China) according to the manufacturer’s protocol[[2](#_ENREF_2)]. pMyc-TA-luc was constructed by using the pGL6-TA plasmid as a template and inserting multiple MYC DNA binding elements at its multiple cloning sites to detect the activation level of MYC with high sensitivity. Plasmids were transfected into cultured cells by Lipofectamine 2000. Then the transfected cells were treated with THZ1.

**Extracellular flux analysis via seahorse metabolic system**

Extracellular flux analysis was performed on the Seahorse XFe24 analyzer. The mitochondrial stress assay and glycolytic stress test were utilized under the instructions by the manufacturer and as described earlier [[3](#_ENREF_3)]. Briefly, 2 x 10^4^ cells were seeded with fresh medium containing drugs. After 24 h treatment, the extracellular acidification rate (ECAR) was measured while cells were exposed to glucose (10 mM), followed by oligomycin (1 M) and completed by 2-DG (50 mM). Oxygen consumption rate (OCR) was detected while cells were exposed to oligomycin (1 M), followed by FCCP (0.5 M) and completed by Rotenone and Antimycin A (0.5 M, respectively).

**Lactate and NAPDH/NAPD+ measurements**

Cells were plated in 6-well plates at a density of 1 x 10^5^ cells per well and allowed to adhere overnight. After treatment for the indicated time, lactate content was analyzed in the cell culture medium using the lactate assay kit (Sigma-Aldrich, USA) according to the manufacturer’s instructions. Treated cells as above were lysed in the NADP extraction buffer supplied with the NADP/NADPH assay kit (Beyotime Biotechnology, China). Extracts were analyzed to quantitate the NADPH/NADP+ ratio according to the manufacturer’s instructions. Results were normalized to the control.

**Reactive oxygen species (ROS)**

The intracellular ROS level was probed with H2DCFDA (Invitrogen, USA) according to the manufacturer's instructions. Briefly, treated cells were harvested to make a single cell suspension and incubated with dye for 30 minutes before flow cytometry analysis. Data were analyzed and shown by FlowJo software.

**References**

1. Wang J, Wang Y, Mei H, Yin Z, Geng Y, Zhang T, Wu G, Lin Z: **The BET bromodomain inhibitor JQ1 radiosensitizes non-small cell lung cancer cells by upregulating p21.** *Cancer Lett* 2017, **391:**141-151.

2. Li SG, Shi QW, Yuan LY, Qin LP, Wang Y, Miao YQ, Chen Z, Ling CQ, Qin WX: **C-Myc-dependent repression of two oncogenic miRNA clusters contributes to triptolide-induced cell death in hepatocellular carcinoma cells.** *J Exp Clin Cancer Res* 2018, **37:**51.

3. Zhang Y, Ishida CT, Ishida W, Lo SL, Zhao J, Shu C, Bianchetti E, Kleiner G, Sanchez-Quintero MJ, Quinzii CM, et al: **Combined HDAC and Bromodomain Protein Inhibition Reprograms Tumor Cell Metabolism and Elicits Synthetic Lethality in Glioblastoma.** *Clin Cancer Res* 2018, **24:**3941-3954.
